# Neuro-JEPA: Building and Evaluating Health-System Scale Neuroimaging Foundation Models with Mixture of Experts

**DOI:** 10.21203/rs.3.rs-11130462/v1

**Published:** 2026-10-15

**Authors:** Haoxu Huang, Long Chen, Jingyun Chen, Jinu Hyun, James Loftus, Kara Melmed, Daniel Orringer, Jennifer Frontera, Seena Dehkharghani, Arjun Masurkar, Narges Razavian

## Abstract

Magnetic resonance imaging (MRI) of the brain routinely combines complementary contrasts—including T1-weighted (T1w), T2-weighted (T2w) and FLAIR sequences—to capture anatomy and pathology. Although foundation models trained on large neuroimaging datasets have shown promise, it remains unclear whether they effectively integrate these complementary contrasts across diverse clinical settings. Here we present Neuro-JEPA, a multimodal foundation model that learns unified representations across structural brain MRIs. Neuro-JEPA combines latent predictive learning with a sparse Mixture-of-Experts architecture to encode multiple MRI contrasts within a single model. The model is pretrained on 1.55 million scans from 428,647 clinical imaging studies spanning T1w, T2w and FLAIR sequences. The model performance is evaluated on 47 downstream tasks across three independent health systems and 12 public datasets, encompassing single-contrast, multimodal and cross-domain applications. Across these evaluations, Neuro-JEPA achieved the strongest overall performance and generalized robustly across heterogeneous cohorts, outperforming existing neuroimaging foundation models and strong conventional baselines in the majority of comparisons despite using less than one-third of the pretraining data of leading models. Performance gains were particularly pronounced in data-scarce settings, reaching up to 15% over prior foundation models for clinically important pathologies including gliosis and multiple sclerosis. Neuro-JEPA showed clinically relevant capabilities across domains, including prediction of amyloid status and progression from mild cognitive impairment to Alzheimer’s disease dementia, stroke identification and classification, and prediction of post-stroke functional outcome and length of stay. Together, these results show that data scale, curation and principled model design can act synergistically to produce broadly transferable neuroimaging representations, and establish Neuro-JEPA as a scalable framework toward general-purpose multimodal foundation models for neurological imaging.

### Introduction

Neuroimaging is central to the care of the patients with neurological conditions, with a multitude of clinical guidelines framed directly around the imaging manifestations of diseases. Magnetic Resonance Imaging (MRI) remains the favored approach for in-vivo tissue characterization, owing to the richness of biological contrasts afforded by carefully tuned acquisition parameters, yielding variable contrast weightings designed to accentuate desired structural, functional, or pathologic features. MRI has thus emerged as an indispensable tool for diagnosis, prognosis, and treatment monitoring for a vast array of neurological conditions, ranging from neurodegenerative diseases [1] and brain tumors [2] to cerebrovascular events [3] and traumatic injuries [4]. With nearly 40 million neuroimaging examinations performed annually in the United States alone [5], the staggering volume of data requiring interpretation by highly specialized medical imaging specialists has spurred demand for scalable computational tools capable of imaging interpretation and decision-support.

Due to the multicontrast nature of brain MRI, comprising potentially dozens of pulse sequences each defined by carefully tuned radiofrequency and magnetic field gradient pulses, even the most routine clinical brain MRI can be viewed as inherently multimodal. These distinct MRI acquisition sequences yield complementary biophysical signals that together inform the interpretation of the exam, each operating along a unique axis sensitized. While considerable variability may exist between brain MRI studies, several pulse sequences stand as essentially standard core contrasts; specifically,T1-weighted (T1w) sequence offers exquisite anatomical contrast; T2-weighted (T2w) sequence are highly sensitized to fluid content; and FLAIR contrasts modifies base T2-weighted contrast through suppression of bulk/free aqueous pools such as the cerebrospinal fluid, in order to increase conspicuity for tissue edema and other pathologies. In clinical practice, expert diagnostic reasoning is informed by the combination of findings across such pulse sequences, which together inform the presence or absence of diseases.

There nevertheless remains a clear gap between this cognitive-clinical exercise and the underlying logic driving contemporary clinical AI paradigms. Most methods operate on a single sequence and cannot exploit cross-contrast synergies, and the multimodal methods that do exist are typically evaluated with all sequences present as input. This leaves two central questions unanswered: does jointly encoding multiple contrasts actually outperform the best single-sequence model, and does any advantage survive under varied disease presentations and differences across sites and scanners? Without controlled unimodal-versus-multimodal comparisons under these conditions, gains attributed to multimodality cannot be distinguished from the benefit of simply having more data at test time. Compounding this, the field lacks principled study of how design choices shape what these models learn, relying instead on direct application of existing algorithms or heuristic modifications. Closing these gaps is essential to move from heuristic design toward a clear understanding of the factors that drive representation learning in human neuroimaging. Under systematic studies on what drives the improved learning of neuroimaging foundation model, our model achieves a large margin of improvement over NeuroVFM [6], despite the latter being pretrained on $\sim$5 million scans (more than three times our pretraining data), underscoring that proper pretraining algorithm, not data scale alone, is decisive for building effective neuroimaging foundation models.

Here we introduce Neuro-JEPA, a sparse multimodal neuroimaging foundation model integrating a Vision Transformer (ViT) [7], joint-embedding predictive learning (JEPA) [8, 9, 10], and a Mixture-of-Experts (MoE) architecture [11, 12, 13, 14, 15]. After pretraining on 1,551,862 MRI sequences (T1w, T2w, FLAIR) from 428,647 studies and 282,693 patients, Neuro-JEPA consistently outperformed frontier neuroimaging foundation models — BrainIAC [16], VoCo [17, 18], and NeuroVFM [6] — across three health systems and twelve public cohorts. Rather than reporting only the all-sequences-present case for each study, we quantify multimodality performance through controlled unimodal, multimodal, and cross-domain comparisons, showing when and by how much joint encoding different modalities helps.

Importantly, Neuro-JEPA extends neuroimaging foundation models to clinically consequential disease settings and endpoints that have received comparatively limited attention in prior work. From routine structural sequences alone, Neuro-JEPA demonstrated strong performance across acute stroke and neurodegenerative disease tasks, including stroke identification and classification, prediction of 90-day modified Rankin Scale (mRS) outcome and hospital length of stay, identification of small or subtle lesions, estimation of cerebral amyloid status from standard T1w, T2w, and FLAIR imaging using amyloid PET as the reference standard, and prediction of progression from prodromal to clinically manifest neurodegenerative disease. These tasks address major unmet clinical needs. Rapid recognition of stroke is essential because ischemia, hemorrhage, and mimics require fundamentally different and often time-sensitive management, while early outcome prediction can inform treatment decisions, recovery expectations, and discharge planning. In Alzheimer's disease, determination of cerebral amyloid burden is increasingly central to diagnosis and eligibility for disease-modifying therapies, yet current approaches rely on PET, cerebrospinal fluid assays, or emerging blood-based biomarkers that may be costly, invasive, or incompletely accessible. More broadly, predicting progression from prodromal to symptomatic stages in disorders such as Alzheimer's and Parkinson's disease could enable earlier

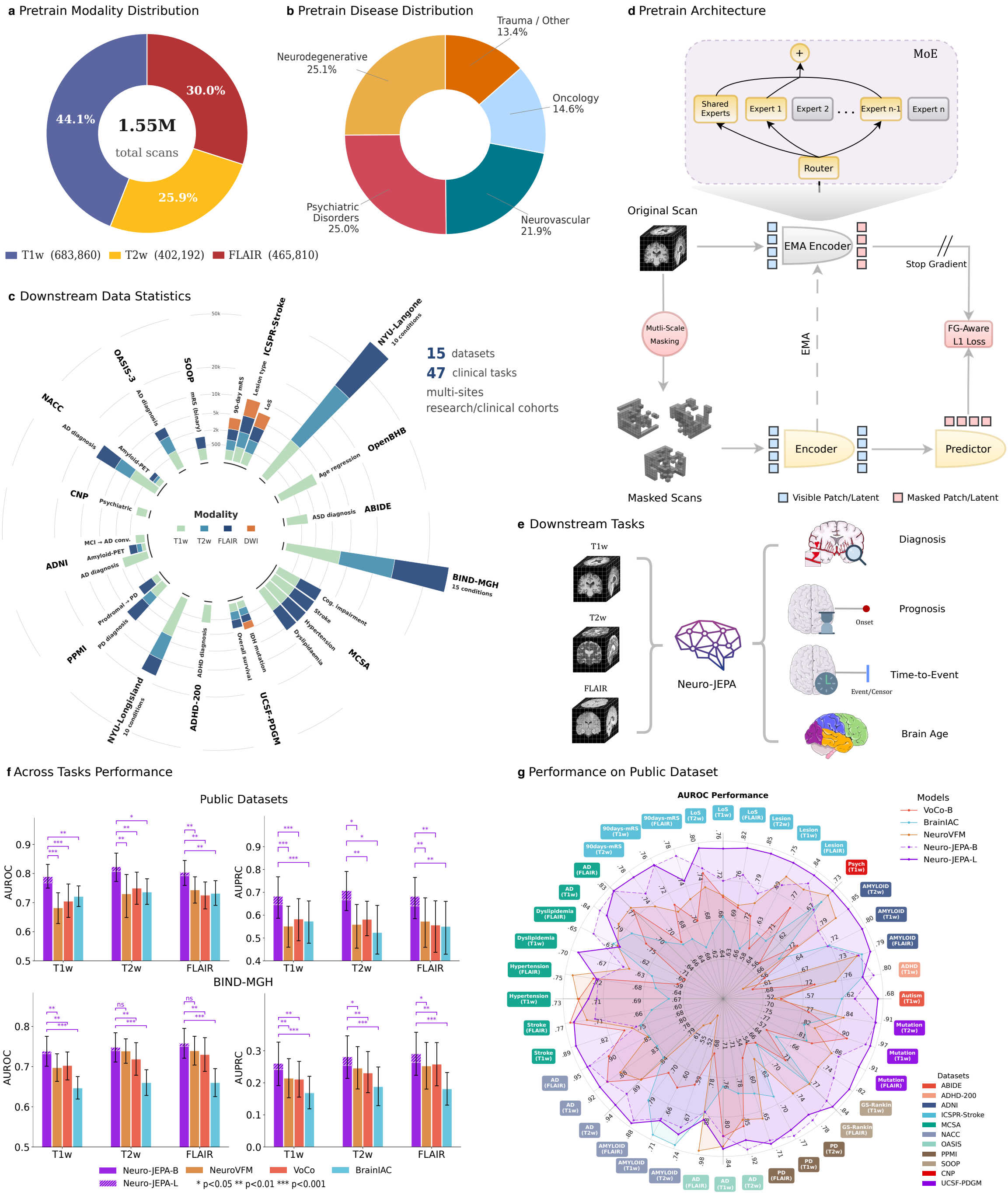

**Figure 1. Overview of the study** - the full pipeline on pre-training Neuro-JEPA with data distribution and performance evaluations. **a,** MRI modalities distribution on T1w, T2w and FLAIR. **b,** Disease distribution on pre-training data from five main categories. **c,** Number of patients for each task and modality on evaluated downstream datasets. **d,** Neuroimaging specialized pre-training architecture built upon JEPA with improvement on masking strategies, foreground-aware loss and backbone sparsification with MoE. **e,** Downstream evaluation with FM-NeuroSp on Diagnosis, Prognosis, Time-to-Event and Age Prediction by taking input from three modalities (T1w, T2w, FLAIR). **f,** Models average performance across tasks for public datasets and BIND-MGH with statistical significance on each modalities (reported on both AUROC and AUPRC). **g,** Per task AUROC performance on different tasks for different foundation models with public datasets. Full name and definition for each task abbreviation is present in Section "Public Research Datasets"

risk stratification, anticipatory guidance, and enrichment of clinical trials targeting disease before substantial irreversible neurodegeneration occurs. Reliable inference of these molecular, diagnostic, and prognostic endpoints from sequences already acquired in routine clinical examinations could therefore extend clinically actionable information to settings where specialized testing is unavailable, delayed, or costly. Together, these capabilities highlight the promise of predictive latent-space foundation models not only for extracting clinically meaningful representations of current disease state, but also for learning trajectories of disease evolution that may ultimately support future-state prediction, longitudinal forecasting, and clinically informed planning.

### Results

Although structural MRI sequences (T1w, T2w, FLAIR) share a common physical origin, their distinct statistical profiles, tissue contrasts, and clinical utilities present learning multi-sequence brain MRI signals as a multi-modal representation learning problem. Under this framing, we developed and validated Neuro-JEPA, a Mixture of Experts (MoE) transformer-based foundation model optimized for neuroimaging, accompanied by detailed analyses of multi-modal learning. Fig. 1 provides an overview of the study and its high-level results. Neuro-JEPA was pretrained on data drawn from the imaging informatics archive of a large health system, spanning diverse disease profiles, demographics, and imaging devices (N=282,693 patients; 1,551,862 multi-modal MRI sequences across T1w, T2w, and FLAIR) (Fig. 1.a and Fig. 1.b), with the characteristics of the training data reported in Supplementary Table 1. The model was trained to handle all three modalities within one architecture. To evaluate its capabilities, we assessed both how well the model encodes individual modality representations and how effectively it captures complementary information across modalities under multi-modal learning. These evaluations span heterogeneous neurological phenotypes and clinical endpoints, including neurodegenerative disease, cerebrovascular disease, inflammatory and structural abnormalities, and both diagnostic and prognostic tasks, enabling us to assess whether a single pretrained representation transfers across clinically distinct settings.

We performed our main evaluations on both the ViT-Base (Neuro-JEPA-B) and ViT-Large (Neuro-JEPA-L) models with MoE (Fig. 1f,g and Fig. 2, with detailed configurations in Section "Model Architecture"). The remaining ablation experiments (Figs. 3 to 5) were conducted using the Neuro-JEPA-B (86 million activated parameters out of 122 million total). This ensures that the number of activated parameters is comparable to the baseline foundation models used in our evaluations for a fair comparison, including BrainIAC (88 million parameters), VoCo-Base (73 million parameters), and NeuroVFM (86 million parameters). Scaling the parameter count via the ViT-Large model with MoE (303 million activated parameters out of 429 million total) presented an average improvement of 1.5–1.9% on AUROC and 1.9–4.1% on AUPRC, as demonstrated in Section "Scalability Under Model Size", representing the effectiveness of our method under model size scaling. All performance comparisons mentioned in the following sections are calculated based on Neuro-JEPA-B model unless otherwise specified.

Across these evaluations, Neuro-JEPA achieved strong performance, with average improvements of 4.4–6.4% in AUROC and 6.4–9.4% in AUPRC over state-of-the-art alternatives (Fig. 1.f,g; Section "Uni-Modal Learning Capability"; Fig. 2). Importantly, these gains extended across clinically distinct disease domains and endpoint types rather than being concentrated within a single disease category. In neurodegenerative disease, Neuro-JEPA performed strongly in predicting amyloid status and progression from mild cognitive impairment to Alzheimer's disease dementia, and demonstrated competitive performance for Parkinson's disease. In cerebrovascular disease, the model supported both stroke identification and classification and prognostic endpoints, including 90-day modified Rankin Scale and length of stay. Performance gains were also pronounced for disorders including gliosis and multiple sclerosis, demonstrating transfer across heterogeneous neurological phenotypes.

To quantify multimodal gains, we measured the added-value in downstream task performances, comparing the best multi-modal trained model vs. the best uni-modal trained alternative for each task. Neuro-JEPA exhibits consistent higher improvements in uni- vs multi-modal setting, and strong overall multimodal performance compared to existing foundation models with 5.8–7.6% improvement on AUROC and 6.2–8.5% on AUPRC with evaluated public datasets (Section "Multi-Modalities Learning Capability", Fig. 3).

We further investigated the design of self-supervised pretraining within the latent predictive modeling paradigm. We demonstrate that direct deployment of existing configurations, such as V-JEPA 2 [9], yields suboptimal performance on neuroimaging. Through systematic analysis and extensive experimentation, we identified three critical adaptations for robust and improved pretraining: (1) multiscale masking, which optimally calibrates the difficulty of the anatomical prediction task; (2) MoE-driven sparsity, which scales model capacity while disentangling heterogeneous neuroimaging anatomies and (3) background signal suppression, which forces the predictive loss to prioritize brain tissue. Comprehensive evaluations of these design choices are detailed in the Results (Section "Key Architectural Components for Effective Pre-training") and Methods (Section "Model Architecture").

Beyond full-data evaluations, we assessed adaptation under limited supervision [16, 19, 20], a property of particular importance in neuroimaging, where expert annotations and disease-specific cohorts are frequently scarce. Neuro-JEPA consistently outperformed all comparison models across few-shot settings, with the largest gains observed for selected clinical phenotypes: AUROC improvements of up to 15% over prior best neuroimaging foundation models for gliosis and multiple sclerosis, and of more than 5% for several clinically important conditions, including Alzheimer's disease, IDH mutation status, and cancer (Section "Label Efficiency Under Few Shots"; Fig. 5). These results indicate that the learned representations remain transferable even when task-specific supervision is limited.

We next asked whether the benefits of Neuro-JEPA extended beyond comparisons among pretrained foundation models. Each foundation model was therefore compared with a neuroimaging-specific CNN trained from scratch for the corresponding downstream task [21]. Across public datasets, Neuro-JEPA was the only evaluated foundation model to outperform the supervised CNN baseline on average, whereas other foundation models showed inconsistent advantages over task-specific supervised learning (Section "Comparison with a supervised CNN baseline"). This result indicates the importance of holistic neuroimaging foundation model evaluation beyond pretrained counterparts.

Finally, we assessed scaling with respect to pretraining data size using 10%, 30% and 100% of the training corpus. Under controlled model architectures, downstream performance improved monotonically with increasing pretraining data (Section "Scalability Under Pretraining Data Size"). Together with the architectural ablations and model-size scaling experiments, these findings show that Neuro-JEPA benefits from both increased data and model capacity.

### Uni-Modal Learning Capability

To assess the performance of individual modality encoding, we benchmarked on diagnosis and prognosis tasks with Neuro-JEPA across 105 dataset–task–modality combinations spanning 3 institutions and several publicly available research-grade datasets. Our study datasets include NYU Langone (NYU) and NYU Long Island (LI) with 10 EHR-driven detection tasks for Hematoma subtypes, Cancer, Edema, Dementia, Major Depressive Disorder, and Hydrocephalus; Massachusetts General Hospital (MGH) across 15 radiology-driven outcomes: Edema, Cyst, Enhancement, Hematoma, Infarct, Atrophy, Ischemic, Mass Effect, Multiple Sclerosis, Midline Shift, Glioblastoma Multiforme, Gliosis, Astrocytoma, and Schwannoma. In addition, we evaluated model quality on 12 research (public) datasets derived from large international studies of Dementia and Aging (ADNI, NACC, OASIS, and MCSA), Parkinson's Disease (PPMI), Stroke (SOOP and ICSPR-Stroke), Glioblastoma (USCF-PDGM), Attention-Deficit/Hyperactivity Disorder (ADHD-200), Autism Spectrum Disorder (ASD), Neuropsychiatric Phenomics (CNP) and Age prediction (OpenBHB). For each of these tasks and datasets where T1w, T2w or FLAIR modalities were present, we assessed performance of our model with that modality. Our MoE model is a single unified architecture capable of representing any (T1w, T2w, FLAIR) modalities, and we evaluated it via fine-tuning for each representative task/data/modality. Neuro-JEPA consistently outperformed three state-of-the-art neuroimaging foundation models (BrainIAC, VoCo, and NeuroVFM) across all settings (Figs. 1 and 2; Extended Data Fig.1 and 2). The detailed averaged unimodal model performance across the tasks and datasets with corresponding 95% confidence intervals is reported in Supplementary Table 9.

On 41 dataset–task–modality combinations from public research-grade datasets, Neuro-JEPA improved mean AUROC by 4.4–6.4% and mean AUPRC by 6.4–9.4% over all baselines (all $p < 0.001$), outperforming other models by the majority of individual comparisons (AUROC: wins 32–35 out of 41 combinations; AUPRC: 31–37 out of 41 combinations). Advantages were substantially larger on internal institutional cohorts. On NYU Langone (30 combinations), Neuro-JEPA improved AUROC by 5.3–9.5% and AUPRC by 11.4–16.1%, with improvement on nearly all tasks (AUROC: wins 29–30 out of 30 combinations; AUPRC: wins 29–30 out of 30 combinations; all $p < 0.001$). Equivalent improvements were observed on NYU Long Island (30 combinations; AUROC: +6.7 to +13.2% and wins 28–30 out of 30 combinations; AUPRC: +8.8 to +17.1% and wins 27–30 out of 30 combinations; all $p < 0.001$). On BIND-MGH (45 combinations), Neuro-JEPA retained consistent advantages (AUROC: +1.6 to +8.5%, wins 34–44 out of 45 combinations; AUPRC: +1.6 to +7.4%, wins 29–45 out of 45 combinations), though improvements over NeuroVFM were more modest (AUROC $p < 0.001$; AUPRC $p = 0.011$).

Across 6 time-to-event dataset–task–modality combinations, Neuro-JEPA achieved improved concordance indices

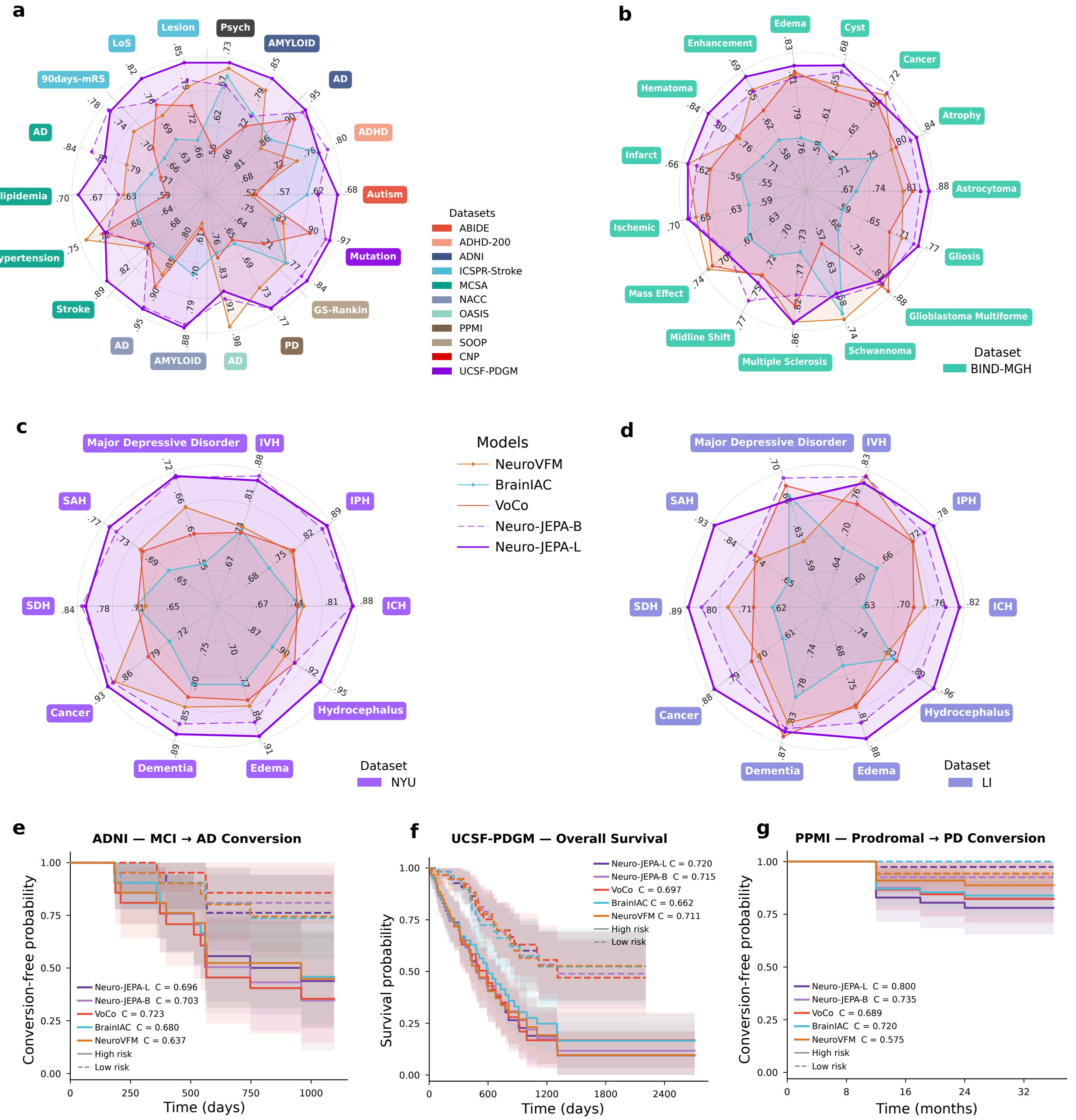

**Figure 2.** **Best Achievable Unimodal Performance** - AUROC for diagnosis/prognosis and C-index for time-to-event tasks are reported with performance from best modality across four foundation models (NeuroVFM, BrainIAC, VoCo and Neuro-JEPA) evaluated under full finetuning except NeuroVFM. The result shows that our model achieves overall best performance across the tasks. **a,** per task performance on best performance modality per task on public datasets. **b,** per task performance on best performance modality per task on BIND-MGH. **c,** per task performance on best performance modality per task on NYU Langone. **d,** per task performance on best performance modality per task on NYU Long Island. **e,** time-to-event on Mild Cognitive Impairment (MCI) to Alzheimer Dementia (AD) conversion with ADNI dataset. **f,** time-to-event on overall survival (until death) on Gliobastoma with UCSF-PDGM dataset. **g,** time-to-event on Prodromal to Parkinson (PD) conversion with PPMI dataset.

(C-index) relative to BrainIAC (+4.5%; 95% CI: 2.7–6.3; $p < 0.001$; wins 6 out of 6 combinations), NeuroVFM (+6.6%; 95% CI: 2.7–12.2; $p < 0.001$; wins 6 out of 6 combinations), and VoCo (+3.1%; 95% CI: 0.6–5.4; $p = 0.015$; wins 5 out of 6 combinations; Extended Data Fig.3.

On the OpenBHB brain-age prediction benchmark ($n = 757$ for test set), Neuro-JEPA attained $R^2 = 0.894$, MAE = 2.78 years, and RMSE = 4.15 years (Extended Data Fig.4). These metrics surpassed BrainIAC ($\Delta$MAE = −2.64 years [1], 95% CI: 2.37–2.93; $\Delta R^2 = +0.372$, 95% CI: 0.326–0.413; both $p < 0.001$), NeuroVFM ($\Delta$MAE = −1.58 years, 95% CI: 1.35–1.75; $\Delta R^2 = +0.221$, 95% CI: 0.182–0.249; all $p < 0.001$), and VoCo ($\Delta$MAE = −3.44 years, 95% CI: 2.90–3.92; $\Delta R^2 = +0.783$, 95% CI: 0.752–0.795; both $p < 0.001$).

Collectively, these results demonstrate that Neuro-JEPA learns robust, transferable unimodal representations that generalize across diverse neuroimaging tasks, imaging modalities, and clinical populations.

### Multi-Modal Learning Capability

Multimodal integration is known to yield nuanced benefits: prior work has identified cases in which multimodal fusion provides marginal gain over unimodal baselines [22, 23, 24] and in which performance is highly sensitive to the selected fusion strategy [25, 26], reflecting differences in how models organize modality-specific representations in latent space. Despite these well-characterized challenges, existing multimodal foundation models have not been systematically evaluated for their multimodal learning capacity. We therefore conducted a comprehensive benchmark spanning five fusion strategies and diverse modality combinations across the evaluated models (Fig. 3;Extedned Data Fig.5-6 and Supplementary Figs. 7 to 9), with mean multimodal performance averaged across tasks and datasets with corresponding 95% confidence intervals reported in Supplementary Table 10.

We assess multimodal capability using two complementary criteria. First, a robust multimodal model should achieve consistently strong performance across tasks under best performing multimodal fusion method. Second, effective multimodal learning should yield positive cross-modal transfer, whereby joint modeling improves performance relative to unimodal baselines (defined as the difference between best achievable multimodal and unimodal performance). For multimodal combination selection, we designate T1w as the structural anchor modality and assess multimodal learning by incorporating complementary contrasts from T2w and FLAIR sequences. This design yields two targeted experimental settings (T1w+T2w and T1w+FLAIR), enabling systematic evaluation of how distinct and clinically relevant signal complements contribute to representation learning. Across both criteria, Neuro-JEPA demonstrates improved performance compared with existing approaches (as demonstrated in Fig. 3, Extended Data Fig.5-6, and Supplementary Figs. 7 to 9), indicating that it captures complementary information across modalities in a stable and generalizable manner. Notably, Neuro-JEPA demonstrates consistent positive transfer across nearly all evaluated tasks in the clinical cohorts from the BIND-MGH dataset, highlighting its strong multimodal capability when deployed in real-world clinical settings.

On public-dataset multimodal tasks (12 combinations), Neuro-JEPA outperformed all baselines. Relative to BrainIAC, Neuro-JEPA improved AUROC by 7.6% (95% CI: 4.7–10.2; $p < 0.001$; wins 12 out of 12 combinations) and AUPRC by 8.5% (95% CI: 4.9–12.0; $p = 0.0015$; wins 11 out of 12 combinations). Relative to NeuroVFM, improvements were 5.8% on AUROC (95% CI: 2.9–9.4; $p = 0.014$; wins 9 out of 12 combinations) and 6.2% on AUPRC (95% CI: 0.7–10.3; $p = 0.005$; wins 9 out of 12 combinations). Relative to VoCo, Neuro-JEPA improved AUROC by 6.2% (95% CI: 3.8–8.0; $p < 0.001$; wins 12 out of 12 combinations) and AUPRC by 7.5% (95% CI: 3.3–10.6; $p < 0.001$; wins 9 out of 12 combinations).

Consistent advantages were observed on BIND-MGH multimodal tasks (30 combinations). Relative to BrainIAC, Neuro-JEPA improved AUROC by 7.8% (95% CI: 6.5–9.4; $p < 0.001$; wins 28 out of 30 combinations) and AUPRC by 9.2% (95% CI: 7.1–11.5; $p < 0.001$; wins 30 out of 30 combinations). Relative to NeuroVFM, improvements were 2.1% on AUROC (95% CI: 1.2–2.8; $p < 0.001$; wins 25 out of 30 combinations) and 3.9% on AUPRC (95% CI: 2.0–6.6; $p < 0.001$; wins 24 out of 30 combinations). Relative to VoCo, Neuro-JEPA improved AUROC by 3.4% (95% CI: 2.7–4.3; $p < 0.001$; 28/30 combinations) and AUPRC by 5.3% (95% CI: 3.5–7.8; $p < 0.001$; wins 29 out of 30 combinations).

Collectively, the results confirmed that Neuro-JEPA excels in both multimodal evaluation criteria across all tested configurations. It achieves superior task performance under optimal fusion and consistently exhibits better positive multi-modal transfer relative to unimodal baselines in comparison to other evaluated models.

[1] $\Delta$ represents absolute difference

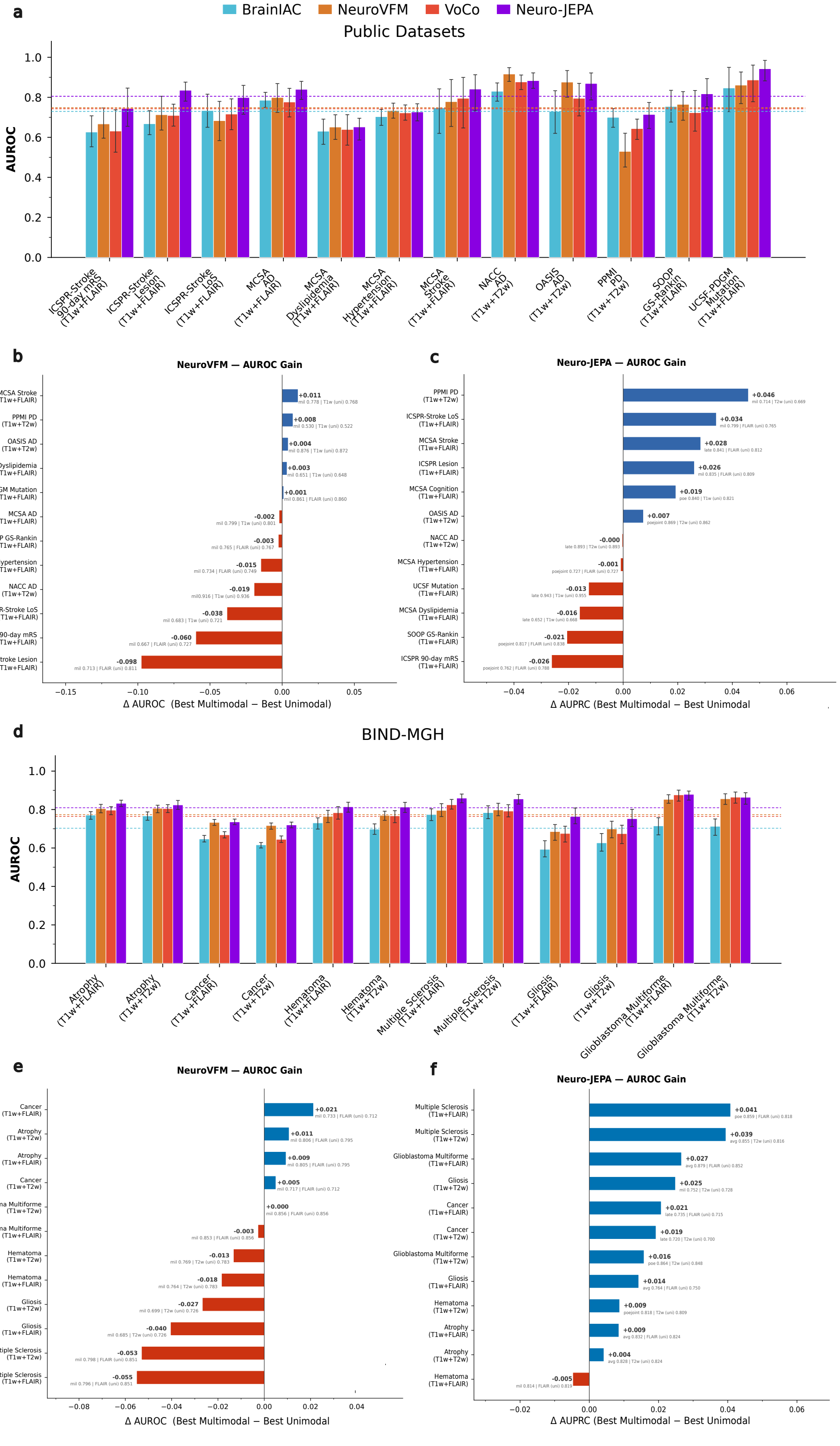

**Figure 3.** **Multimodal Performance and Gain over Unimodal Baselines.**

**Fig. 3 (continued).** We report AUROC for paired multimodal inputs and the corresponding multimodal gain, defined as the difference between the best multimodal fusion result and the best unimodal result. For fair comparison, unimodal baselines in this analysis were re-trained on the subset of cases with complete modalities availability on multimodal counterparts and Neuro-JEPA-B is used in the evaluation. Additional AUPRC results and comparisons with other evaluated models are provided in Supplementary Figs. 5, 6 and 18 to 21. **a,d,** AUROC of multimodal fusion across selected public datasets and BIND-MGH for four foundation models. For each model, performance is reported using the best fusion strategy among five evaluated methods. Dotted lines indicate mean performance across tasks, showing that Neuro-JEPA outperforms competing foundation models by a substantial margin. **b,c,e,f,** AUROC gain from multimodal fusion for NeuroVFM, the best previous foundation model, and Neuro-JEPA on public datasets and BIND-MGH. Neuro-JEPA yields overall higher and more consistent gains.

### Components for Effective Pre-training

We identified three key components improving the generalizability of JEPA pre-training in neuroimaging. First, masking strategy determines the quality of the predictive learning signal. Second, sparse computation via Mixture-of-Experts (MoE) architectures enhances the model's capacity to disentangle heterogeneous anatomical tokens by routing information through different pathways. Third, suppressing learning signals from background regions improves robustness, as 40–60% of voxels in skull-stripped neuroimages are non-informative. Full implementation details are provided in Model Architecture and Supplementary Section B.

To quantify the contribution of each component, we performed controlled ablation studies using the V-JEPA 2 pre-training framework as baseline, incrementally introducing: (1) multi-scale masking, (2) multi-scale masking with MoE, and (3) multi-scale masking with MoE and a foreground-aware $L_1$ loss to downweight background voxels (down-weighting ratio $\beta = 0.1$). Each modification yielded consistent performance gains on attentive probing averaged across evaluated datasets on NYU Langone, NYU Long Island and Massachusetts General Hospital (Fig. 4). Multi-scale masking alone improved mean AUROC by 1.5% and AUPRC by 2.5% ; adding MoE contributed a further 3.9% (AUROC) and 3.4% (AUPRC); incorporating the foreground-aware $L_1$ loss added 0.9% (AUROC) and 2.8% (AUPRC). More details on per dataset and modality performance on 30% pretrain data are present in Supplementary Fig. 22.

### Label efficiency Under Few Shots

We evaluate model label efficiency under few-shot conditions in Fig. 5. The result is evaluated with $k = \{16, 32, 64, 128, 256\}$ and with fine-tuning on attentive layers, where $k$ is the number of positive samples used for classification and number of samples used for each quartile of the original age distribution for age prediction. The result is presented on four selected tasks on public datasets, NYU-Langone and BIND-MGH with AUROC average across all modalities. More diverse tasks evaluation on AUROC, AUPRC and per modality performance can be found in Supplementary Figs. 37 to 44. The results demonstrate that Neuro-JEPA achieves superior label efficiency, consistent across all evaluated tasks and particularly for tasks derived from clinical datasets such as NYU-Langone and BIND-MGH. Across different values of k, several tasks showed improvements exceeding 5% in both AUROC and AUPRC, including gliosis, multiple sclerosis, and hematomas. The label-efficiency gains were especially pronounced in tasks for which performance approached saturation under full-data training. For example, on NACC AD, Neuro-JEPA achieved approximately 15% higher AUROC and AUPRC across different k values, despite all compared models attaining close performance when trained on full samples.

### Cross-cohort and out-of-distribution modality generalization

We assessed whether Neuro-JEPA generalizes across independent clinical cohorts and neuroimaging modalities. For cross-cohort evaluation, models were fine-tuned on a source cohort and evaluated without further adaptation on an external target cohort with matched label definitions. This setting approximates direct cross-institutional deployment, in which a model optimized at one institution is applied to data from another. As shown in Supplementary Fig. 32, Neuro-JEPA maintained stable performance across cohort transfers, including NACC-to-ADNI for Alzheimer's disease and amyloid status prediction, and MGH-to-NYU for hematoma prediction, with no substantial degradation in majority of external test performance.

We further evaluated whether Neuro-JEPA can generalize to neuroimaging modalities not observed during pretrain-

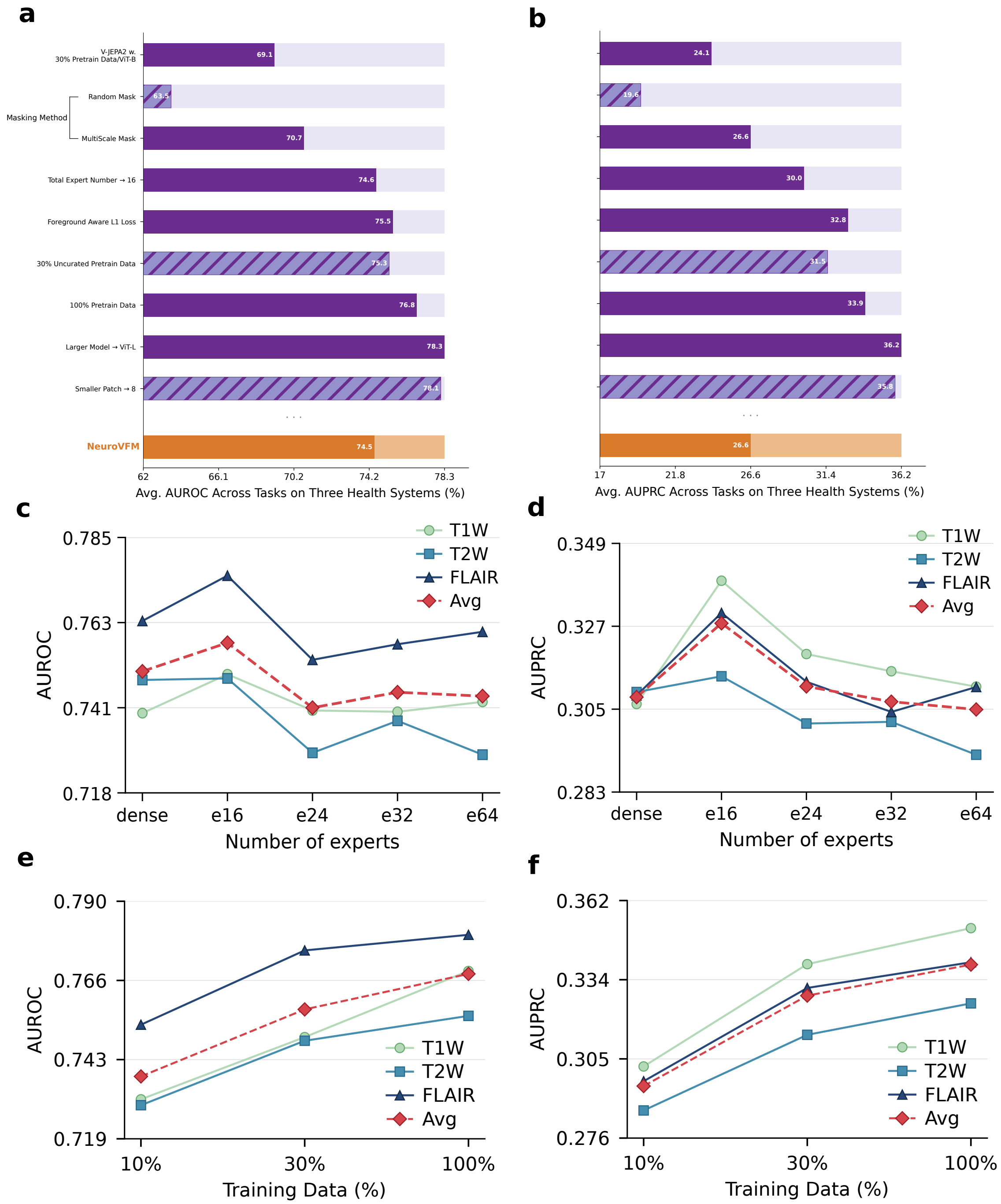

**Figure 4. Ablation of Model Design and Scaling** - AUROC and AUPRC are averaged across all tasks and modalities from three health systems with attentive probing: NYU Langone, NYU Long Island, and MGH. **a,b,** Stepwise ablation of model design [27], in which each modification is introduced sequentially from the original V-JEPA2 implementation to the final largest scale Neuro-JEPA model, showing each design choice brings meaningful contribution to overall performance. Hatched bar means the design choice is not applied. **c,d,** Ablation of the total number of experts, performed with all other design components applied and 30% of pretrain data, showing that increasing the number of experts to 16 improves both AUROC and AUPRC relative to the dense model, whereas further expert increases yield diminishing returns. **e,f,** Ablation of pretraining data scale, showing that performance continues to improve as the proportion of pretraining data increases up to the full dataset.

ing. Specifically, we fine-tuned and evaluated the model on diffusion-weighted MRI (DWI), which was absent from the pretraining data. Experiments were conducted on 90-day modified Rankin Scale (mRS) prediction, lesion type classification, and length-of-stay prediction in ICSPR-Stroke, as well as IDH mutation prediction in UCSF-PDGM. Despite this modality shift, Neuro-JEPA remained the best-performing model among all evaluated baselines, achieving average improvements of 1.7% in AUROC and 1.8% in AUPRC (Supplementary Fig. 33 and Table 11). These findings indicate that Neuro-JEPA learns representations that transfer robustly across both clinical institutions and previously unseen neuroimaging modalities.

#### Scalability Under Pretraining

Scalability is the central principle in the development of foundation models. We evaluate the scalability of our framework on both data and model size. Increasing the pretraining dataset size leads to consistent improvement in downstream performance, with gains of 1.3% in AUROC and 1.1% in AUPRC across evaluation benchmarks shown in Fig. 4, demonstrating effective data scaling. More details on performance change under increased data size can be found in Supplementary Fig. 25. Same as data size scaling, we present study on model size scaling in Supplementary Fig. 26 and Fig. 27, where increasing model size from ViT-B-MoE (121 million total parameters) to ViT-L-MoE (429 million total parameters) effectively improved model performance on both health-system and public datasets with averaged 1.5% improvement on AUROC and 2.3% improvement on AUPRC for clinical datasets and averaged 1.9% improvement on AUROC and 4.1% improvement on AUPRC for public datasets.

#### Comparison with a Simple CNN Baseline

To determine whether foundation-model pretraining provides practical benefit beyond simple supervised learning from scratch, we compared each pretrained foundation model with a neuroimaging-specific CNN trained from scratch for the downstream task [21]. Across 41 task–modality combinations from 12 public datasets, existing foundation models did not consistently outperform this simple CNN baseline, whereas Neuro-JEPA achieved consistent gains, improving average AUROC by 3.7% and AUPRC by 4.5% (Supplementary Table 12; Supplementary Fig. 36). Neuro-JEPA was also the only foundation model to improve over the CNN baseline for age prediction on quasi-raw scans, increasing $R^2$ by $+2.8$ and reducing MAE and RMSE by $-0.37$ and $-0.50$, respectively. These results emphasize that claims of foundation-model superiority should be supported by comprehensive evaluations against simple, task-specific conventional baselines, rather than comparisons limited to other pretrained models.

#### MoE Routing Analysis and Visualization

To evaluate the model's capacity for zero-shot pathological localization, we extracted spatial attention maps by querying patches within a selected lesion-containing region (Fig. 6a). The final maps were computed by weighted averaging (higher weights if the selected patch contain more lesion voxels) the attention scores across these patches. The attention map evaluation is applied to scans exhibiting stroke, multiple sclerosis, and glioblastoma. The samples are selected from datasets with ground truth lesion masks on ATLAS [28], MSLesSeg [29] and UCSF-PDGM [30]. The results demonstrate that strong neuroimaging foundation model intrinsically learned the representation to localizes these diverse pathologies without requiring any disease-specific fine-tuning. Additional visualization result for attention map is present in Extended Fig.8-10.

We analyzed representation feature maps in Fig. 6b by smoothing stacked first three principal components from principal component analysis (PCA). The visualization reveals that representations inherently preserve distinct brain anatomical structures. Additional visualization result for feature map before smoothing is present in Supplementary Fig. 5.

Different tokens routing visualization (Figures 6c-d) and heatmaps of token-level routing distributions (Figures 6e-f); token vs. expert index) confirm that individual experts preferentially route specific token subsets. Projecting averaged routing distributions onto T1w and T2w templates for representative NYU-Langone samples (Figures 6i–j) demonstrates clear spatial segregation, confirming that MoE mechanisms effectively disentangle regional processing by partitioning experts across anatomical structures (see Supplementary Figures 57–60 for extended examples).

Extended analyses further demonstrated foreground–background and cross-modal routing behavior. Most experts displayed pronounced routing imbalances between foreground and background tokens, highlighting strong functional separation (Supplementary Figures I.5–47). Conversely, within foreground regions across T1w, T2w, and FLAIR sequences, experts exhibited largely uniform routing (Supplementary Figures 48–49). This contrasts with previous

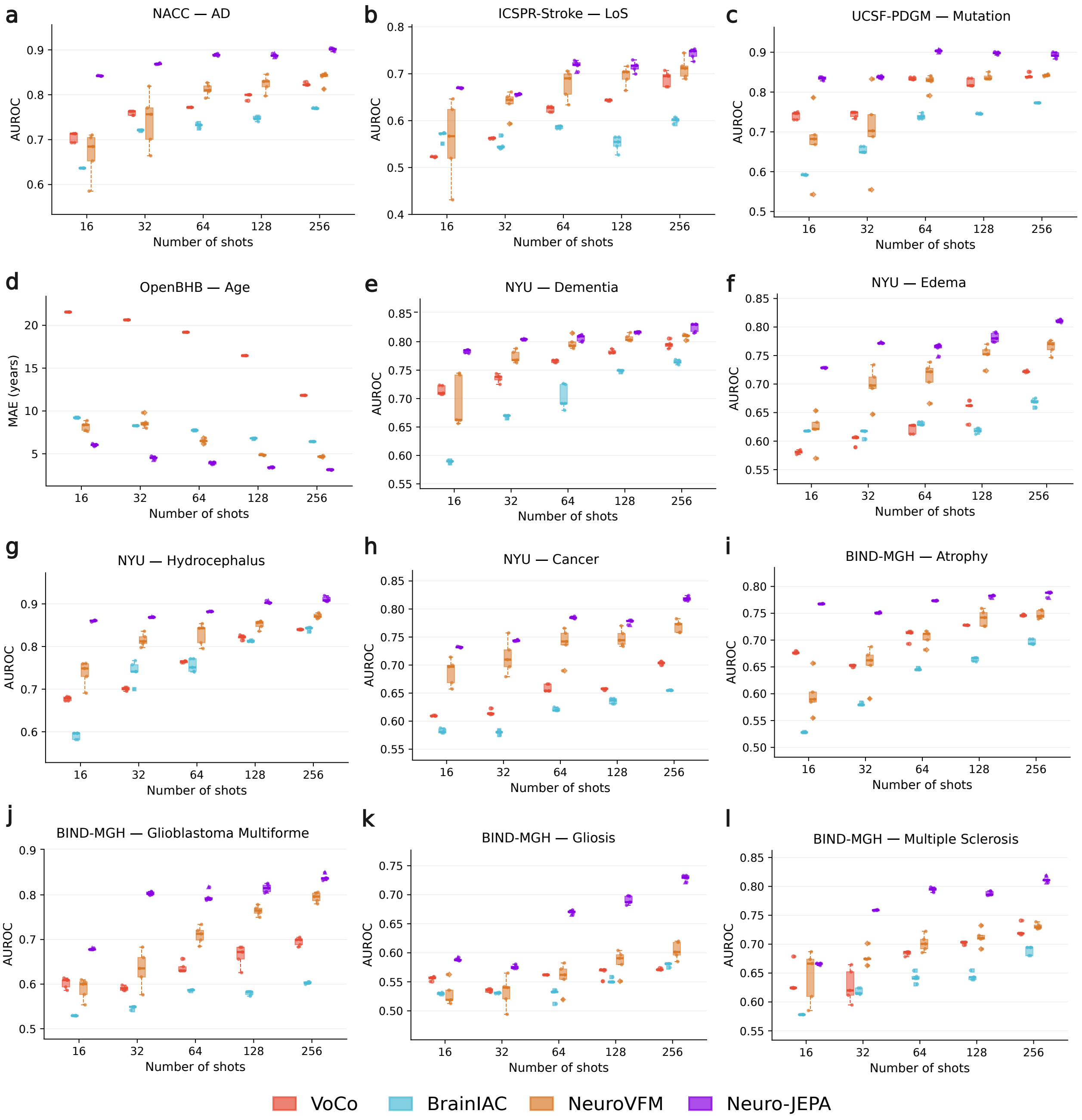

**Figure 5.** **Few-shot Analysis** - we examine the evaluated models label efficiency when only $k = \{16, 32, 64, 128, 256\}$ positive samples are provided on training. Neuro-JEPA-B is used in the evaluation. The performance in reported in AUROC for classification and MAE for regression. The result demonstrates that our model performs better than evaluated models by a large margin under majority of environment with limited labeled data. **a-d,** Few-shot performance on selected tasks from public datasets. All result is reported as averaged performance across all available modalities for each task **e-h,** Few-shot performance on selected tasks from NYU-Langone dataset. **i-l,** Few-shot performance on selected tasks from BIND-MGH dataset.

reports of rigid modality specialization for different experts [14, 31], suggesting instead that the learned experts from Neuro-JEPA leverage high cross-modal anatomical redundancy to disentangle information processing on different shared structural regions.

### Discussion

Modern neurological diagnosis depends on multimodal neuroimaging, which captures complementary biological information needed for clinical decision-making. Although recent multimodal neuroimaging foundation models have shown promise for integrating these signals [6, 32], the effects of pretraining design and the robustness of multimodal representations remain insufficiently understood. Here, we introduce *Neuro-JEPA*, a vision foundation model pretrained on approximately 1.55 million carefully curated brain MRI scans. Across clinical cohorts from three major health systems and research cohorts spanning 12 public datasets, Neuro-JEPA showed strong and consistent performance across unimodal and multimodal tasks, with robust generalization across heterogeneous imaging settings.

Building on the joint-embedding predictive architecture (JEPA), our results show that data scale, curation and model design can act synergistically to improve multimodal representation learning. Joint multimodal pretraining improved not only multimodal fusion but also the quality of unimodal representations, suggesting that learning across complementary imaging sequences yields features that transfer broadly across downstream tasks. Scaling and training-trajectory analyses further showed that Mixture-of-Experts architectures can remain stable during large-scale pretraining. Together with advances in multimodal learning, general-purpose architectures and scalable foundation-model training [31, 33, 34, 35, 36, 37], these findings support continued efforts to combine larger and more diverse datasets with principled architectural and algorithmic development.

The generalist nature of Neuro-JEPA is particularly relevant to clinical neuroimaging, where radiologists must simultaneously evaluate numerous pathologies, anatomical variants and technical artifacts. Building separate specialist models for each potential finding is difficult to scale, requires substantial computational and deployment infrastructure, and is poorly suited to the long-tailed distribution of neurological abnormalities encountered in practice. By contrast, a single pretrained model that transfers effectively across tasks could provide a common foundation for multiple diagnostic and prognostic applications. This is especially important in neuroimaging, where examination volumes continue to increase and interpretation often requires specialized expertise, including for time-sensitive and high-acuity conditions.

Neuro-JEPA also showed complementary strengths across major neurological disease domains. In neurodegenerative disease, it captured clinically meaningful imaging phenotypes associated with amyloid status, progression from mild cognitive impairment to Alzheimer's disease dementia and Parkinson's disease, supporting the potential use of routine MRI for opportunistic detection and longitudinal risk stratification. In stroke, Neuro-JEPA performed strongly in disease identification and classification and also predicted 90-day functional outcome and length of stay, extending its utility from diagnostic recognition to prognosis and care planning. Collectively, these results suggest that broadly transferable neuroimaging representations can support clinically distinct tasks across disease categories without requiring a separately pretrained model for each application.

Several limitations define important directions for future work. Neuro-JEPA was pretrained on three widely used MRI sequences—T1-weighted, T2-weighted and FLAIR—to enable controlled evaluation of multimodal learning. Clinical neuroimaging, however, also relies on diffusion-weighted imaging, susceptibility-weighted imaging, contrast-enhanced MRI, functional MRI and other modalities such as CT, each of which provides complementary pathophysiological information. Incorporating these additional inputs may further expand the clinical scope of general-purpose neuroimaging models. Our experiments also used down-sampled scans and fixed patch sizes to enable systematic ablations under computational constraints. Future work should evaluate higher spatial resolution and alternative patching strategies, which may be particularly important for small or fine-grained abnormalities [9, 38].

Finally, the differences observed between internal and external evaluations highlight the importance of training on broader multi-institutional data. Neuro-JEPA outperformed NeuroVFM on both cohorts, but the margin was substantially larger on the internal NYU cohort than on the external MGH cohort, underscoring the limitations of single-system pretraining and the need for models exposed to greater institutional, demographic and acquisition heterogeneity.

In summary, Neuro-JEPA provides a scalable framework for multimodal brain MRI representation learning and a systematic analysis of how data scale, architecture and pretraining objectives influence downstream performance. Beyond the specific model introduced here, our findings support a broader shift from narrowly specialized imaging

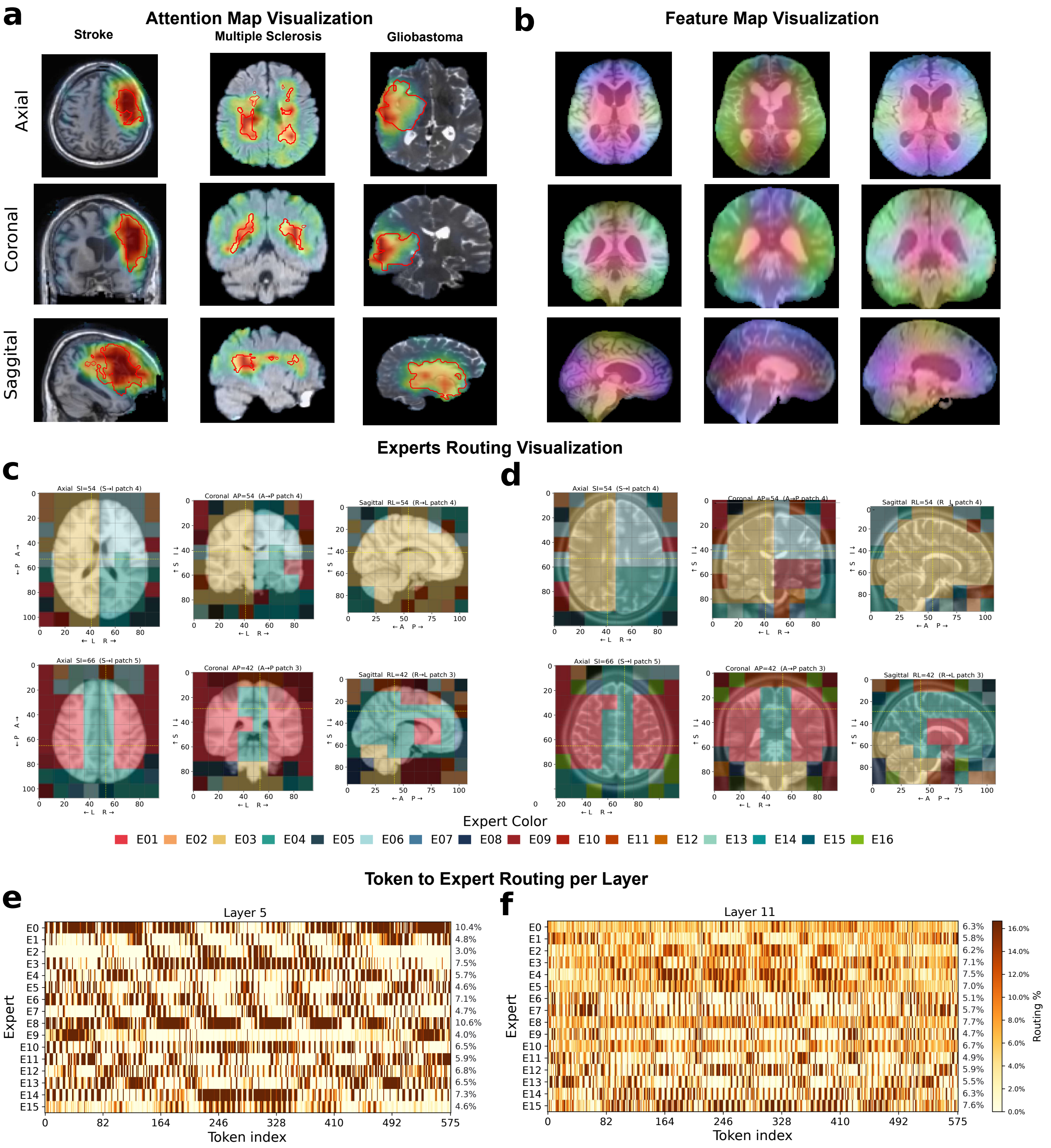

**Figure 6. MoE Routing Analysis and Visualization** - we explore visualization with attention map and feature map from Principal Components Analysis (PCA) to identify if Neuro-JEPA learns meaningful brain structure from pre-training. Then, we explore the MoE routing behavior by examining if different experts are routing to different regions. **a,** Attention map visualization on lesions of Stroke, Multiple Sclerosis and Gliobastoma, where the red circles indicate ground truth lesion masks **b,** Feature map on selected of pre-training data **c,d,** Routing regions on different experts **e,f,** Token routing frequency on different experts from different token indices.

algorithms toward unified neuroimaging foundation models that can transfer across modalities, diseases and clinical tasks. Continued expansion to more diverse imaging sequences, institutions and patient populations will be essential to determine whether such models can ultimately support robust, generalizable and clinically useful AI systems for neurological imaging.

## Methods

### Datasets

#### *Data Curation*

Registered MRI scans are used for all cohorts, where all brain MRI scans used for pretraining and downstream evaluation were affine registered to the MNI152 standard space and resampled to an isotropic resolution of $1.0 \times 1.0 \times 1.0$ mm$^3$ using FSL [39]. T1w and T2w sequences were registered to their corresponding MNI152 templates, whereas FLAIR sequence were registered to the MNI152 T2w template because of their closer contrast characteristics. We further applied bias-field correction and skull stripping with SynthStrip [40], which also facilitated robust anonymization by removing non-brain tissue. Similar registration strategies have been used in recent brain MRI modeling studies [16, 41].

Clinical MRI cohorts acquired directly from hospital environments often contain artifacts that degrade image quality, including severe noise, signal dropout, and partial or near-complete loss of brain tissue, as illustrated in Supplementary Fig. 30. These artifacts are primarily attributable to patient-related factors, such as motion during acquisition. Although large-scale foundation models are often expected to acquire robustness from heterogeneous data, consistent with observations in other domains on the role of data quality in foundation model training [42, 43, 44], our empirical analysis indicated that severely degraded scans can compromise pretraining efficacy. Specifically, we conducted an ablation study whereby 25,000 previously excluded low-quality scans were reintroduced into a 30% subset of the pretraining data, corresponding to approximately 5% of the full pretraining corpus, reducing model performance as shown in Fig. 4a,b.

To mitigate the effects of severe image degradation, we implemented a quantitative quality-control pipeline after image registration. For each registered scan, we computed Mutual Information (MI), Peak Signal-to-Noise Ratio (PSNR), and Pearson correlation with the corresponding MNI152 reference template. Scans were excluded if they satisfied any of the following modality-specific criteria: for T1w, MI $< 0.30$, PSNR $< 10$, or Pearson correlation $< 0.85$; for T2w, MI $< 0.30$, PSNR $< 11$, or Pearson correlation $< 0.65$; and for FLAIR, MI $< 0.25$, PSNR $< 11$, or Pearson correlation $< 0.65$. These thresholds were selected by manually reviewing the lowest-quality scans within each modality and identifying values that removed images with severe tissue loss or prohibitive noise while retaining scans with only mild quality degradation. This curation procedure reduced the pretraining data from 1,639,685 to 1,551,862 scans, corresponding to the removal of approximately 5% of the data. We further confirmed that this data-curation step improved model performance, as shown in Fig. 4a,b and Supplementary Fig. 29.

#### *Data Split*

We split all datasets at the patient level such that the training, validation, and test sets contain non-overlapping individuals. Although some previous studies [6, 32] have adopted time-based splits, reserving future time points for evaluation, this design still permits the same patient to appear in both training and evaluation sets, thereby introducing substantial risk of data leakage and overly optimistic performance. We therefore adopted patient-level splitting to provide a more rigorous assessment of representation generalizability.

This choice is motivated by the following considerations. Medical imaging models are well-known to exploit spurious correlations or direct data leakage at the patient level [45, 46, 47, 48] and high-dimensional 3D scans often contain highly individual-specific anatomical patterns that can function as biometric-like signatures. For example, when the same patient appears in both training and evaluation sets, it is known that models can rely on patient identity instead of disease-relevant imaging features [49, 50] to perform diagnostic tasks. This issue can easily inflate model performance even without robust representation learning on disease related features. Additionally, patient-level splitting ensures a consistent evaluation framework across all datasets, where the temporal information for the public datasets is often intentionally anonymized by date shifting such as BIND-MGH and several clinical trial cohorts in our evaluation benchmark.

#### *Pretraining Dataset*

The pretraining dataset is collected from NYU Langone Picture Archiving and Communication System (PACS) system. After data curation, the pretrain dataset consists of 1,551,862 scans across 282,693 patients and 428,647 studies with all scans performed between 2009 and 2025. We partitioned the full dataset by the patient IDs into training and held-out downstream evaluation sets to avoid the leakage of scans from the sample patients. This yielded 88,314 (35,183 unique patients) samples for held-out downstream evaluation, although the model was only pretrained on the 1,551,862 scans from the training set.

#### *Downstream Datasets*

For unimodal experiments, we leveraged the full set of available samples for each dataset and task. For multimodal evaluation, we restricted analyses to the subset of samples with all modalities (T1, T2, FLAIR) availability. This design ensures balanced contributions from each modality during both training and evaluation, enabling a controlled and unbiased assessment of the difference between unimodal and multimodal performance. By standardizing modality availability, we minimize confounding effects that could otherwise arise from unequal sample distributions across modalities and bias comparative analyses.

Our downstream benchmark spans a diverse set of clinical and research cohorts, including two internal hospital systems from distinct sites (NYU Langone and NYU Long Island), one geographically distinct external institution (Massachusetts General Hospital), and 12 high-quality public research datasets. Together, this collection represents, to our knowledge, one of the most comprehensive and heterogeneous benchmarks in the neuroimaging foundation model literature for evaluation of model generalizability across clinical sites, research cohorts, and imaging contexts. Detailed descriptions of dataset composition and downstream tasks are provided below. Multimodal evaluation was performed for datasets with sufficient overlap in samples across modalities, enabling controlled assessment of multimodal representation learning. Full dataset statistics are provided in Supplementary Table 3.

##### Health System Datasets

**NYU Langone Hopsitals Manhattan and Brooklyn — 10 tasks** We used data from the NYU Langone main campus (Manhattan and Brooklyn) for internal in-domain (ID) evaluation (abbreviated as NYU Langone). Disease labels were derived from electronic health records (EHR) within a 3-month window centered on the imaging date, based on ICD-10 diagnostic codes and medication records (Supplementary Table 2). The dataset comprises 19,325 T1w scans from 10,004 patients, 29,237 T2w scans from 19,132 patients, and 23,302 FLAIR scans from 15,937 patients. Data are split at the patient level into training, validation, and test sets (60/20/20). We evaluated 10 clinical conditions. The conditions we examined include Cancer, Edema, Dementia, Major Depressive Disorder, Hydrocephalus (HCP), Intraparenchymal Hemorrhage (IPH), Intraventricular Hemorrhage (IVH), Subdural Hemorrhage (SDH), Subarachnoid Hemorrhage (SAH), Intracerebral Hemorrhage (ICH)

**NYU Langone Hospital Long Island — 10 tasks** We use data from NYU Langone Hospital Long Island as an internal out-of-domain (OOD) evaluation cohort (abbreviated as NYU Long Island), with labels defined identically to NYU Langone. The dataset includes 8,024 T1w scans from 3,928 patients, 5,050 T2w scans from 3,206 patients, and 3,376 FLAIR scans from 3,065 patients. Patient-level splits follow the same 60/20/20 protocol.

**Massachusett General Hospital (MGH) — 15 tasks** We evaluate external generalization using a curated subset of data from the Massachusetts General Hospital [51]. Disease labels are extracted from radiology reports using a large language model (Bio-Medical-Llama-3-8B [52]), achieving $> 90\%$ accuracy as verified by expert manual annotation on a subset. To ensure fair comparisons, we construct balanced subsets with equal sample sizes across modalities for both unimodal and multimodal evaluations. The dataset includes 22,142 scans from 11,802 patients, with patient-level splits of 60/20/20. Fifteen tasks are selected based on label prevalence spanning tumor, vascular, inflammatory, and degenerative conditions. The tasks included Astrocytoma, Atrophy, Cyst, Edema, Enhancement, Hematoma, Infarct, Ischemic, Mass Effect, Midline Shift, Multiple Sclerosis, Schwannoma, White Matter Changes, Glioblastoma Multiforme, Gliosis.

##### Public Research Datasets

**ABIDE — 1 task** The Autism Brain Imaging Data Exchange (ABIDE) [53] aggregates multi-site neuroimaging data across 17 international cohorts. We evaluate binary classification of autism versus Healthy Controls using T1w MRI with 1,099 patients and equal number of samples as patients. Patient-level split is performed on 60/20/20.

**ADHD-200 — 1 task** The ADHD-200 [54] dataset comprises structural MRI from eight international imaging centers. We evaluated ADHD versus Healthy Control classification using T1w scans with 776 patients and equal number of samples as patients. Patient-level split is performed on 60/20/20.

**ADNI — 3 tasks** The Alzheimer's Disease Neuroimaging Initiative (ADNI) [55] is a multi-center longitudinal study across North America. We evaluated (1) Amyloid positivity (ADNI4 dataset), (2) Alzheimer's disease (AD) versus Healthy Control classification (ADNI1 standardized subset is used) and (3) Mild Cognitive Impairment (MCI) to AD conversion as a time-to-event task. For amyloid detection with amyloid positivity determined from PET imaging, The dataset include 167 patients for T1w, 205 patients for T2w and 317 patients for FLAIR. Equal numbers of samples as subjects are presented for amyloid detection. For AD classification, 1632 T1w scans from 455 patients is used. For MCI to AD conversion, 209 T1w scans from 209 patients is used. Data are split at the patient level with 60/20/20.

**ICSPR-Stroke — 3 tasks** The Annotated Clinical MRIs of Patients with Acute Stroke dataset (ICSPR-Stroke) [56] is a high-density, longitudinal repository of 2,888 multimodal MRIs collected at a National Stroke Center. We define three tasks from this dataset. (1) 90-day functional outcome with modified Rankin Scale (mRS) as binary classification (0-2 mRS as 0 and 3-6 mRS as 1);(2) lesion type classification (ischemic, hemorrhagic or absent); and (3) length of stay at abinary classification threshold of 8 days. Because every task has missing labels and modalities, each presents different numbers of patients and samples as detailed in Supplementary Table 3. Only T1w and FLAIR are used for multimodal evaluation. The data are split by 60/20/20 at the patient level.

**MCSA — 4 tasks** The Mayo Clinic Study of Aging (MCSA) [57] is an ongoing, longitudinal population study of residents in Olmsted County, Minnesota, dedicated to mapping the trajectories of cognitive impairment and successful aging. We framed a total of 4 tasks based on the availability of diagnosis labels from this dataset. This includes tasks are diseases vs. Healthy Control with diseases to be AD, stroke, hypertension and dyslipidemia. The dataset includes 2,873 scans from 1,715 patients in total with T1w and FLAIR subsets for unimodal and multimodal analyses. The data are split by 60/20/20 at the patient level.

**NACC — 2 tasks** The National Alzheimer's Coordinating Center (NACC) [58] dataset is among the world's largest and most comprehensive longitudinal repositories for Alzheimer's disease and related dementias (AD/ADRD). NACC aggregates and standardizes multimodal data from over 40 Alzheimer's Disease Research Centers (ADRCs) across the United States. We evaluate Alzheimer's disease versus control and amyloid status prediction across modalities. For AD vs. Healthy Control under unimodal evaluation, it presents 4,994 total samples from 3,841 patients for T1w, 3,062 total samples from 2,538 patients for T2w and 3,755 samples from 3,024 patients for FLAIR. For Amyloid vs. Non-Amyloid PET positivity classification task, it presents 182 samples from 176 patients for T1w, 97 samples from 92 patients for T2w, 159 samples from 155 patients for FLAIR. For multimodal evaluation, it presents 3,772 total samples from 3,132 patients for T1w and T2w. The data are split by 60/20/20 at the patient level.

**OASIS3 — 1 task** The Open Access Series of Imaging Studies (OASIS3) [59] is a open-science repository providing three decades of longitudinal clinical, cognitive, and multimodal neuroimaging data. The data is collected from Washington University in St. Louis (Knight Alzheimer Disease Research Center). Our main focus was on classifying AD vs. Healthy Control for this dataset. For unimodal evaluation, it presents 1,924 total samples from 1,126 patients for T1w, 1,665 total samples from 1,004 patients for T2w and 1,028 samples from 727 patients for FLAIR. For multimodal evaluation, it presents 1,384 samples from 883 patients for T1w and T2w. The data are split by 60/20/20 at the patient level.

**PPMI — 2 task** The Parkinson's Progression Markers Initiative (PPMI) [60] is a international observational study designed to identify and validate robust clinical, imaging, genetic, and biochemical biomarkers of Parkinson's disease (PD) progression with data collected from nearly 50 international sites. Our focus was on (1) discriminating Parkinson's vs. Prodromal vs. Healthy Controls for this dataset (2) determining conversion from Prodromal to Parkinson as a time-to-event task. For Parkinson diagnosis, under unimodal evaluation, it presents 1,763 total samples from 1,000 patients for T1w, 1,296 samples from 346 patients for T2w and 2,365 samples from 1,577 patients for FLAIR. Under multimodal evaluation, it presents 1,339 samples from 302 patients for T1w and T2w. For Prodromal to Parkinson conversion, it presents 397 total samples from 397 patients for T1w, 882 samples from 882 patients for FLAIR. The data are split by 60/20/20 at the patient level.

**SOOP — 1 tasks** The Stroke Outcome Optimization Project (SOOP) [61] dataset is a large-scale neuroimaging repository comprising acute clinical MRI scans and linked metadata collected in South Carolina. For this dataset, we evaluated functional outcome as modified Rankin Scale at patient discharge as binary classification (denoted as GS-Rankin for SOOP) with 0-2 as 0 and 3-6 as 1). It includes 647 patients with equal numbers of samples as patients. T1w and FLAIR are used in both unimodal and multimodal evaluation. The data are split by 60/20/20 at the patient level.

**CNP — 1 task** The Preprocessed Consortium for Neuropsychiatric Phenomics (CNP) [62] dataset is a rigorously curated neuroimaging resource designed to accelerate the study of brain-behavior relationships across the neuropsychiatric spectrum, encompassing healthy controls as well as individuals diagnosed with adult ADHD, bipolar disorder, and schizophrenia. We performed a three-way classification defined by healthy control, ADHD, and bipolar disorder or schizophrenia using T1w. It presents 265 patients with equal number of samples as patients. The data are split by 60/20/20 at the patient level.

**UCSF-PDGM — 2 task** The University of California San Francisco Preoperative Diffuse Glioma MRI (UCSF-PDGM) [30] dataset is an open-access radiogenomic repository in neuro-oncology, focused on Gliobastoma. We evaluated IDH mutation status prediction and overall survival as a time-to-event task. The data includes 495 total samples from 295 patients, wherein all samples have equal number of T1w, T2w and FLAIR. The data are split by 60/20/20 at the patient level.

**OpenBHB — 1 task** OpenBHB is a large-scale, multi-site brain MRI dataset designed to support robust brain-age modeling and evaluation under acquisition-site variability. The dataset aggregates 5,330 three-dimensional T1w brain MRI scans from healthy controls across 10 publicly available cohorts, covering 71 acquisition sites and participants from European-American, European and Asian populations. Becuase ages were not made available for the original test set, we split the original training set by 80/20 producing 2581 patients for training and 646 patients for validation. The original validation set is used as the test set with 757 patients. In order to evaluate the model performance under minimal preprocessing, we used quasi-raw (preprocessed with bias-field correction, affine registration to MNI space) scans in all evaluations.

### Model Architecture

#### *Backbone Model*

Motivated by previous studies, demonstrating strong model performance and scalability of 3D Vision Transformers (ViT) on volumetric data such as video [9] or medical imaging [63], we employed 3D ViT with Mixture of Experts (MoE) architecture as our backbone. Specifically, the 3D ViT backbone of Neuro-JEPA model includes 12 transformer layers, each based on 768 hidden dimensions, 3072 dimension feedforward layers, and 12 attention heads. To introduce sparsity, MoE routing is enabled on alternating layers (6 MoE layers out of the 12 total) [14]. The MoE configuration utilizes 2 shared experts and 16 total experts, with 6 experts activated per forward pass, softmax scoring gating and a routing scaling factor of 4.0 (detailed ablation studies on these MoE design choices are provided in Supplementary Section I). For positional encoding, we applied 3D Rotary Position Embedding (RoPE) following [9]. Input data from cropped standard templates are resized from $180 \times 216 \times 180$ to $100 \times 120 \times 100$ and then cropped to $96 \times 108 \times 96$. A patch size of $12 \times 12 \times 12$ is used, yielding 576 tokens per scan.

#### *Joint Embedding Pretraining*

Our primary pretraining framework is built upon the Joint-Embedding Predictive Architecture (JEPA). We selected JEPA over alternative methods due to its rapid convergence (fewer than 200 epochs in our experiments) and high throughput, achieved by avoiding the computationally expensive 3D image interpolation required by frameworks like the DINO family [38, 64, 65]. This efficiency enabled training on our complete dataset of approximately 1.5 million scans within one week under constrained computational resources. Additionally, we showed that this approach outperforms alternative reconstruction based methods (e.g. Masked Autoencoder) under equivalent settings as demonstrated in Supplementary Fig. 31. Fundamentally, JEPA predicts the latent representations of masked regions based on visible context. The masked volume is processed by the online encoder, while the full volume target is generated by a momentum encoder updated via an Exponential Moving Average (EMA) of the online encoder's weights (see Figure Fig. 1 for an architectural overview). JEPA has recently demonstrated strong scalability and performance across diverse data in 3D domains, including video [9], physical planning [66], echocardiography [67], and neuroimaging [6, 68].

Because our pretraining data underwent skull stripping and defacing, approximately $40-60\%$ of each resulting volume consists of background. To prevent the model from overfitting to low-information background signals while preserving essential spatial anchoring, we modified the standard $L_1$ loss in JEPA into a foreground-aware $L_1$ loss. Specifically, we down-weight the loss contributed by predicted background regions, which we define as voxels with intensities outside the 2nd–98th percentile range of the current scan. Applying an intensity-based cutoff incurs negligible computational cost in comparison to calculating the foreground with a segmentation algorithm. For a batch of $B$ samples (indexed by $b$) and $M$ masks, where each mask $m$ contains $K$ tokens (indexed by $k$) with predicted embedding $\hat{y}$ and target embedding $y$, the loss is formulated as:

$$L = \frac{1}{B \cdot M \cdot K} \sum_{b,m,k} w_{b,m,k} \cdot \left| \hat{y}_{b,m,k} - y_{b,m,k} \right|$$

where the normalized weight as

$$w_{b,m,k} = \frac{w'_{b,m,k}}{\frac{1}{K} \sum_{k=1}^{K} w'_{b,m,k}}$$

and the unnormalized weighting function as

$$w'_{b,m,k} = \beta + (1-\beta) f_{b,m,k}$$

on the foreground map $f \in [0,1]$ (i.e. when $f = 1$ (Foreground), The weight becomes 1.0. When $f = 0$ (Background), the weight reduces to $\beta$). The normalization is important in this setup because it ensures that the average weight per mask is always 1.0, regardless of how much background is in the image. In our experiment, $\beta$ is set to be 0.1. Full details on pretraining hyperparameter setting is present in Supplementary Section B.6.

##### ***Mixture of Experts***

Introducing sparsity is crucial for efficient learning representations [11, 12, 13, 14, 15]. We systematically evaluated the role of sparsity in building a multimodal neuroimaging foundation model by integrating MoE. Our design incorporates 2 shared experts and routes tokens to 6 active experts out of 16 total, using a routing scale of 4.0 approximated via sampling [69]. This sparsity rate is intentionally lower than that of some recent Large Language Models (LLMs) based MoE models [31, 70, 71], as our ablation studies in Fig. 4c,d indicate diminishing performance returns with further sparsification. We apply MoE on every other layer for feedforward layers in attention blocks instead of in every layer, following [14] towards saving computational cost.

Mixture-of-experts (MoE) models are prone to expert collapse, in which routing concentrates on only a few experts [15]. This is conventionally mitigated by load balancing, a regularization applied to the routing distribution. Here we adopt auxiliary-loss-free bias-update for load balancing [72], which achieves performance comparable to traditional auxiliary-loss methods [11]. We found, however, that the standard auxiliary-loss-free formulation produced unstable load balancing in our setting. To stabilize training, we introduce three modifications. First, token-averaged error correction divides the error correction by the average number of tokens per expert, so that experts further from a balanced distribution receive proportionally larger bias updates. Second, zero-mean projection is applied to the bias terms to prevent unbounded drift. Third, bias clipping constrains bias values that exceed a predefined threshold (set to 0.3 for softmax gating). Together, these modifications reliably prevent expert collapse during JEPA pretraining with MoE under our configuration. The complete modified bias-update algorithm is provided in Supplementary Algorithm 2.

##### ***Multiscale Masking***

Different masking strategies can impose different generalization behaviors on JEPA pre-training as demonstrated from previous works [68, 73]. We empirically observed that the original masking strategy utilized in V-JEPA 2 [9] yields suboptimal performance across our benchmarks. The original masking strategy was designed for video, where strong temporal continuity and redundancy permit the removal of large spatiotemporal blocks. In volumetric neuroimaging, however, such aggressive masking along the depth axis may be detrimental, as it can obscure high-frequency spatial details required to encode small lesions and subtle anatomical variation. We therefore hypothesized that neuroimaging requires a masking strategy that better preserves fine-grained three-dimensional structure. As shown in Fig. 4a,b, random masking patterns that permit easy interpolation between masked targets and visible context substantially degrade downstream performance. Consequently, we propose a modified multiscale masking strategy optimized for masked latent representation prediction for 3D neuroimaging. To reduce reliance on such local interpolation while retaining sufficient depth context for fine-detail prediction, we randomly sample masking blocks across multiple spatial scales until the target masking ratio is reached. Specifically, each mask is iteratively sampled with a spatial scale drawn from one of three ranges, $[0.0, 0.2]$, $[0.2, 0.5]$, or $[0.5, 0.7]$, and a depth scale drawn from the range of $[0.0, 1.0]$, until the fixed masking ratio of 0.75 is achieved. The complete multiscale sampling algorithm is provided in Supplementary Section B.1.

### Evaluation Setting

**Baseline Comparisons** We benchmark our proposed model against VoCo [17, 18], a self-supervised model pre-trained on computed tomography (CT) volumes with overall high performance across neuroimaging, and two domain-specific neuroimaging foundation models: BrainIAC [16] and NeuroVFM [6]. VoCo is a self-supervised learning model designed to enhance 3D medical image analysis by leveraging the consistent geometric relationships of human anatomy. The model is pre-trained on a diverse collection of 160,167 unannotated 3D CT volumes collected from public datasets with SwinUNETR [74] as its model backbone. Its training objective predicts crop positions as pseudo-labels under the assumption of anatomical consistency. The base model is used for VoCo across all evaluations to align parameter sizes. BrainIAC is a 3D brain MRI foundation model pre-trained on 48,965 diverse scans using the contrastive self-supervised framework SimCLR [75] with a Vision Transformer (ViT) backbone to learn universal anatomical representations. NeuroVFM utilizes a ViT backbone, train with JEPA objective on latent masked prediction on only foreground tokens with approximately 5.24 million scans on both MRI and CT volumes. Variable length flash attention [76] is used for handling varying batch lengths per sample for foreground tokens. Although PRIMA [32] is also a relevant baseline, both PRIMA and NeuroVFM were pretrained on similar data from the University of Michigan Health System. We therefore selected a single representative model from this shared pretraining source to avoid redundant comparisons. NeuroVFM was chosen because its image-only latent predictive framework is more closely aligned with our method, enabling a more controlled and informative comparison.

**Unimodal Encoding Evaluation** A robust multimodal foundation model must first demonstrate high-fidelity encoding of individual modalities. Consequently, we establish our baseline by evaluating unimodal encoding performance. We independently encode each modality (T1w, T2w, and FLAIR) and assess the performance of these representations across various downstream tasks with full fine-tuning on attentive layers. Specifically, we evaluated the model across three institutional datasets (NYU Langone, NYU Long Island, and MGH) alongside 12 public datasets. The experimental results are detailed in Figs. 1 and 2; Extended Data Fig 1 and 2, and Supplementary Section C.1.

**Multimodal Fusion Evaluation** Beyond robust unimodal encoding, an effective multimodal model must successfully leverage complementary information when distinct modalities are combined. We evaluated multimodal fusion by comparing the performance of combined modalities against their unimodal counterparts, ensuring all models are fine-tuned on an identical number of samples. This controlled setting prevents the introduction of confounding variables, offering a more rigorous comparison than evaluating against models trained with missing modalities. We selected 12 tasks from 7 public datasets based on sample size of available modalities and evaluated a total of 15 tasks using MGH as an external clinical cohort.

As shown in previous studies [26, 77, 78], achieving optimal multimodal learning requires tailoring fusion methods to specific models and task combinations to effectively capture cross-modal interactions. Accordingly, we applied five distinct fusion strategies and performed comprehensive hyperparameter sweeps for each task and model. Specifically, our evaluation incorporates late fusion with cross-attention, logit averaging, multiple instance learning-based fusion [6], product-of-experts fusion, and inter-intra modality modeling [79] (detailed in Supplementary Section B). As T1w serves as the primary structural reference, while T2w and FLAIR sequences provide distinct complementary contrasts, we evaluate multimodal learning under two clinically grounded settings: (1) T1w+T2w, which integrates anatomical information with broad fluid-sensitive contrast, and (2) T1w+FLAIR, which combines anatomy with lesion-accentuated, cerebrospinal fluid-suppressed contrast. These configurations capture two clinically relevant forms of complementarity, enabling a robust assessment of the multimodal learning quality across different foundation models.

To quantify the benefits of multimodal fusion over unimodal approaches, we compared multimodal gain as defined by the difference between best achievable performance across all fusion methods and the optimal unimodal performance. Comprehensive results detailing individual fusion strategies and modality-specific performance are provided in Supplementary Figs. 5 to 9.

**Few-shots Evaluation** We evaluate model performance under limited data regimes with $K = 16, 32, 64, 128, 256$, wherein the data is sampled such that positive and negative samples are equal to $K$. For age prediction, $K$ present number of samples bootstrapped from each quartile of the full age distribution in the training data. Because the performance for few-shots is highly dependent on the bootstrapped samples, we run each experiment five times on different bootstrapped samples and calculate the mean and 95% confidence intervals. The detailed few-shot evaluation results across datasets are presented in Fig. 5 and Supplementary Figs. 37 to 44.

**Design Choices Evaluation** We investigate three main architectural adaptations to optimize the Joint-Embedding Predictive Architecture (JEPA) for neuroimaging tasks: integrating Mixture of Experts (MoE) across varying expert counts, adopting multiscale masking over standard strategies, and suppressing loss signals from background predictions. Additionally, we show that pretraining scales effectively with model size, whereas decreasing patch size yields no further improvement. The detailed design choice evaluation is presented in Fig. 4a-d and Supplementary Figs. 22 and 23.

**Scaling Analysis** We assessed the efficacy of our pre-training framework on data scaling regimes. The main performance improvements were driven by data scaling and algorithmic improvement, although we did additionally explore parameter scaling. By analogy to the Chinchilla scaling laws [80] and related literature [81, 82], we hypothesize that our current volume of pre-training data does not yet saturate the capacity of our model's parameter space. We empirically validate this conclusion by evaluating model performance when pre-training only on $10\%, 30\%, 100\%$ of the available data as shown in Fig. 4e,f and Supplementary Fig. 25.

#### Statistical analysis

Model performance metrics are reported as the empirical mean alongside 95% confidence intervals (CIs). CIs were estimated via non-parametric bootstrapping of the held-out validation set ($B = 1000$ resamples). To account for the sample-selection variance inherent to few-shot learning, training and evaluation procedures were repeated across five independent random samplings of the training distribution; aggregate means and corresponding CIs are reported. Statistical significance of performance differences between compared models was assessed using a two-sided paired permutation test ($N = 1000$ permutations).

#### Computing Hardware Software

All experiments are performed in PyTorch (v2.10.0), MONAI (v1.5.1), Numpy (v1.26.4), Pandas (v2.2.3), NiBabel (v5.3.2), Omegaconf (v2.3.0), Hydra (v1.3.2). All plots and figures were created by Matplotlib (v3.10.1), Seaborn (v0.13.2) and Plotly (v6.6.0). We customized the implementation starting from original V-JEPA 2 original implementation `https://github.com/facebookresearch/vjepa2`. For evaluation environment on compared models (VoCo — `https://github.com/Luffy03/VoCo`, BrainIAC — `https://github.com/AIM-KannLab/BrainIAC`, NeuroVFM — `https://github.com/MLNeurosurg/neurovfm`), we used the environment provided from their respective Github repositories. All downstream experiments were conducted on a single 80GB NVIDIA A100 GPU (graphics processing unit) or 48GB NVIDIA L40S GPU. All pre-training experiments on ViT Base were conducted on eight/twelve 48GB NVIDIA L40S GPUs in two/three nodes. All data storage and model development were performed on the NYU Langone UltraViolet High Performance Computing Core.

#### Data Availability

The internal clinical data used in this study are unavailable due to privacy restrictions and institutional policy. The public datasets can be obtained by direct downloading or sending requests to the corresponding studies as follows.

ABIDE (`https://fcon_1000.projects.nitrc.org/indi/abide/`), ADHD-200 (`https://fcon_1000.projects.nitrc.org/indi/adhd200/`), ADNI (`https://adni.loni.usc.edu/`),

ICSPR-Stroke (https://www.icpsr.umich.edu/web/ICPSR/studies/38464), MCSA (https://www.mayo.edu/research/centers-programs/alzheimers-disease-research-center/research-activities/mayo-clinic-study-aging/overview), NACC (https://www.naccdata.org/), OASIS3 (https://sites.wustl.edu/oasisbrains/home/oasis-3/), SOOP (https://www.nature.com/articles/s41597-024-03667-5), CNP (https://openneuro.org/datasets/ds000030/versions/1.0.0), UCSF-PDGM (https://www.cancerimagingarchive.net/collection/ucsf-pdgm/), OpenBHB (https://baobablab.github.io/bhb/dataset), and BIND-MGH (https://bdsp.io/content/n1vba1x5qt62frfjem65/1.0/).

The data splits for all our evaluation on public datasets are available in our public code repository.

### Data and Weight Availability

All code required for model pre-training, downstream fine-tuning, and evaluation is publicly available at https://github.com/NYUMedML/Neuro-JEPA. Pretrained model weights are made publicly available upon reasonable request via Hugging Face at https://huggingface.co/NYUMedML/Neuro-JEPA. The model weights are distributed for non-commercial, non-derivatives, non-clinical, academic research purposes under the Creative Commons Attribution-NonCommercial-NoDerivatives 4.0 International License (CC BY-NC-ND 4.0).

### Acknowledgements

Our study was supported by various funding sources. H.H, L.C, A.M. and N.R. are supported by National Institute of Health National Institute on Aging study R01AG085617. A.M. and N.R are also supported by National Institute of Health National Institute on Aging award P30AG066512 and R01AG079175. We acknowledge resources and support from NYU Langone High Performance Computing team, and DataCore which undertook our PACS data request. The authors would like to thank constructive suggestions and feedback from Artie Shen, Carlos Fernandez-Granda, Divyam Madaan, Sumit Chopra and Un J. Kang.

### Author contributions statement

H.H. and N.R. conceived the study. H.H. developed the methodology, implemented the framework, conducted experiments, collected public datasets, preprocessed, analyzed and interpreted the data, and wrote the original manuscript. L.C. assisted with data preprocessing and organization. J.C. instructed data preprocessing pipeline. J.H. assisted on data visualization and model evaluation verification. J.L., J.M., D.O., J.F., S.D., A.M. instructed evaluation protocols and data processing. N.R. collected NYULH data, and supervised the project. All authors contributed to manuscript editing and discussion.

**Extended Data Fig. 1. Per Task AUPRC Across Public Dataset Tasks and AUPRC for Best Achievable Unimodal Performance.** AUPRC for public datasets on each unimodal performance and best achiable AUPRC performance across datasets and tasks. The result shows that our model demonstrates consistent performance improvement in comparison to other foundation models on AUPRC. **a,** AUPRC performance on different tasks with different modalities on public datasets. **b,** AUPRC performance for tasks with best achievable modalities on public datasets. **c,** AUPRC performance for tasks with best achievable modalities on BIND-MGH dataset. **d,** AUPRC performance for tasks with best achievable modalities on NYU Langone dataset. **e,** AUPRC performance for tasks with best achievable modalities on NYU Longisland dataset.

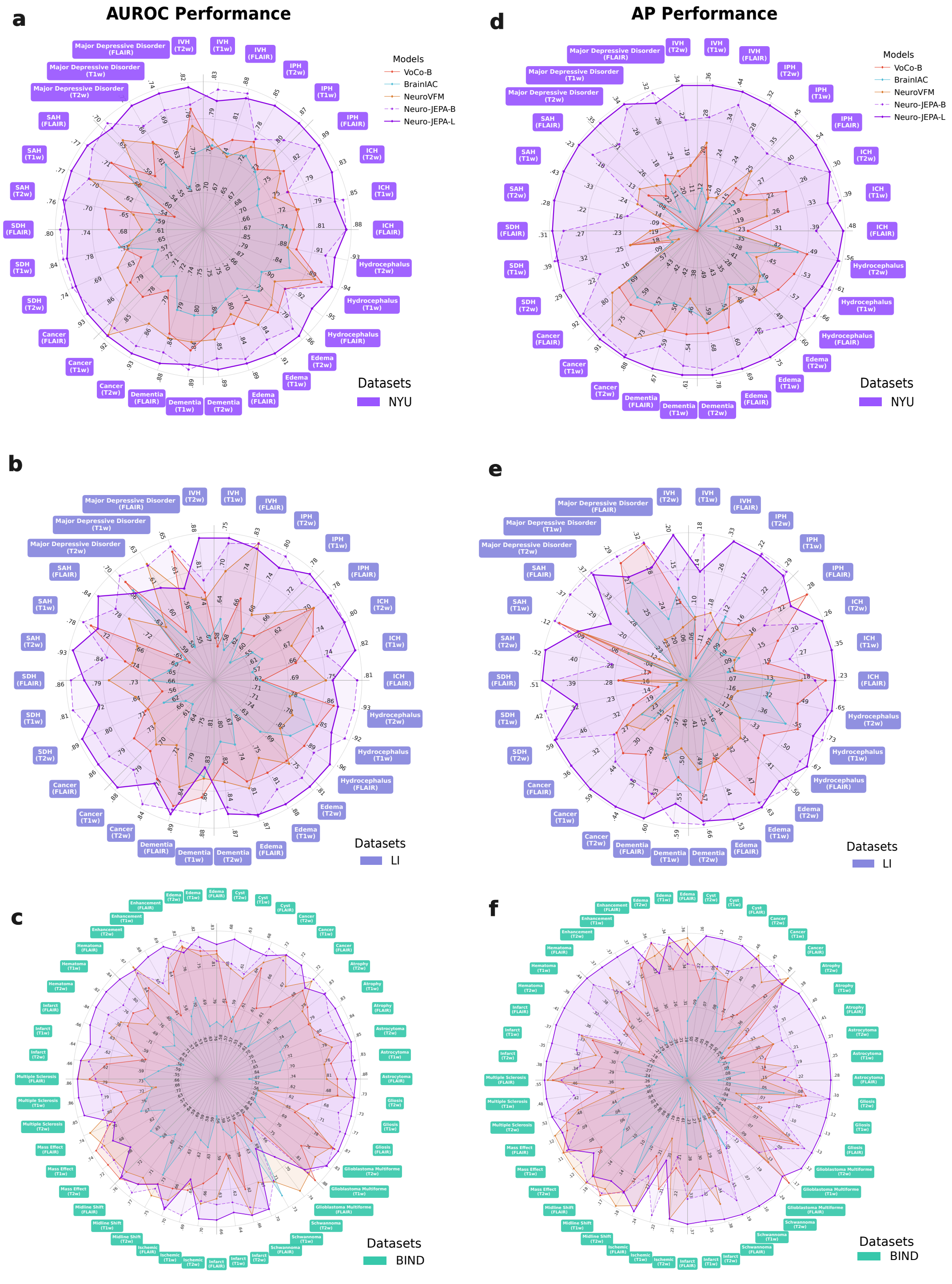

**Extended Data Fig. 2.** **Unimodal Per Task AUROC and AUPRC Across Three Health System Datasets.** AUROC and AUPRC with per modality performance for each task on NYU Langone, NYU Longisland and BIND-MGH datasets across all evaluated foundation models. All tasks are evaluated by full fine-tuning. Our model show improved performance across majority of tasks on different modalities **a,** AUROC for NYU Langone dataset. **b,** AUROC for NYU Longisland dataset. **c,** AUROC for BIND-MGH dataset. **d,** AUPRC for NYU Langone dataset. **e,** AUPRC for NYU Longisland dataset. **f,** AUPRC for BIND-MGH dataset.

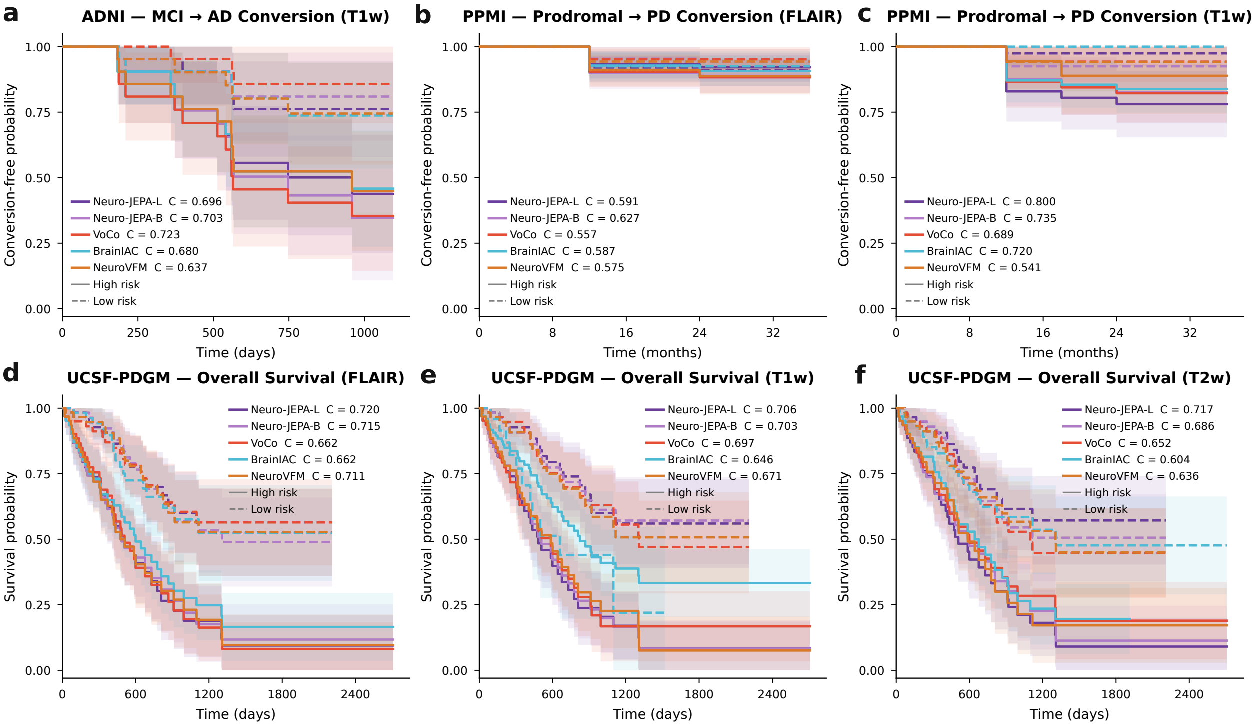

**Extended Data Fig. 3. Kaplan Meier Curve for Time-to-Event on All Modalities.** result is reported with Concordance Index (C-index) Prodromal to PD conversion for PPMI dataset and Overall Survival for UCSF-PDGM dataset. **a,** MCI to AD conversion within 3 years for ADNI dataset with T1w. **b,c,** Prodromal to PD conversion within 3 years for PPMI dataset with T1w and FLAIR. **e-g,** Overall Survival for UCSF-PDGM dataset with T1w, T2w and FLAIR.

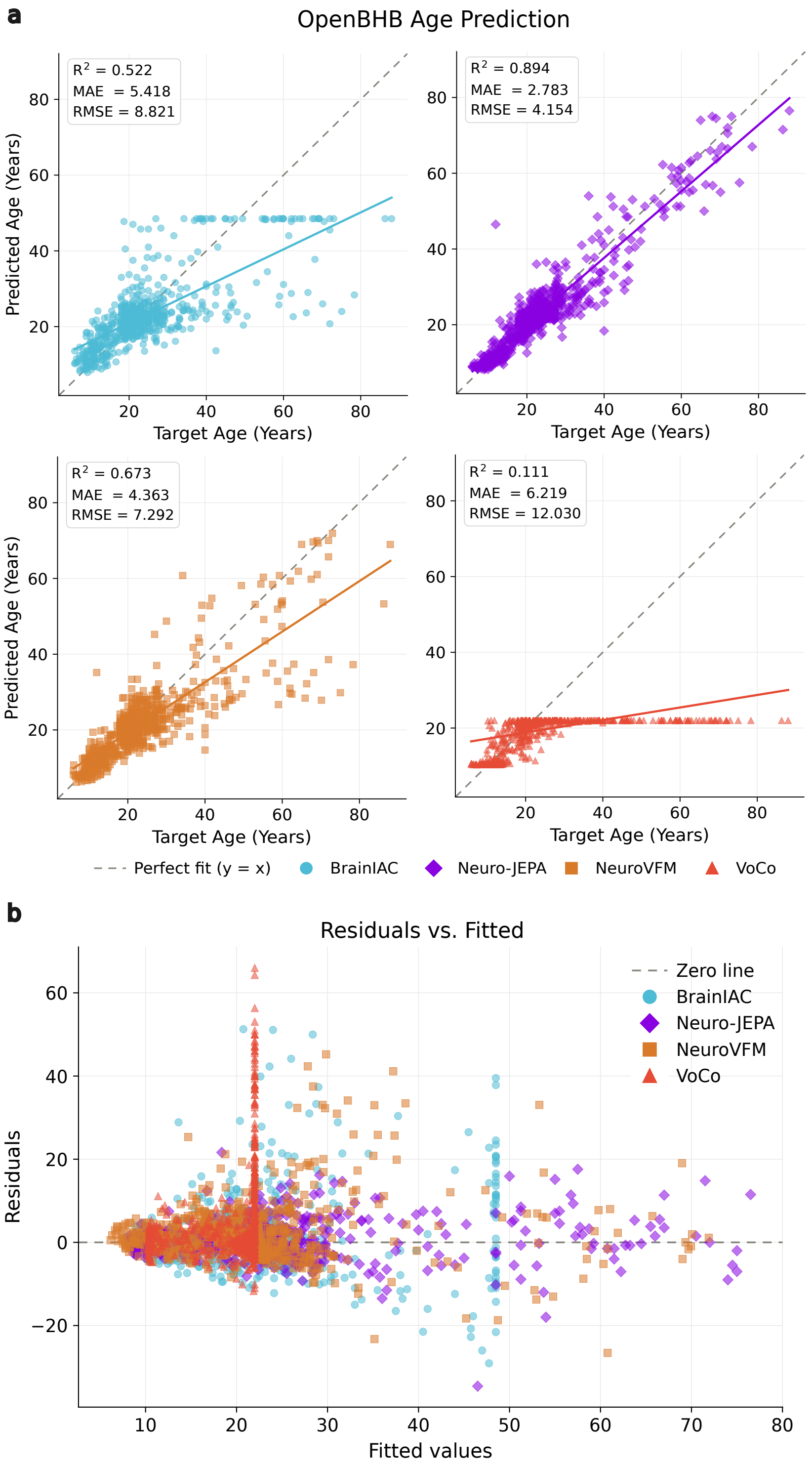

**Extended Data Fig. 4. Age Prediction Comparison on OpenBHB Dataset.** Age prediction as regression on OpenBHB dataset on Quasi-Raw T1w scans with performance reported on $R^2$, Mean Absolute Error (MAE) and Rooted Mean Squared Error (RMSE). The result shows that our model outperform existing foundation models especially with stronger fitting on patients with older age. **a,** regression goodness of fit for each individual foundation models. **b,** residuals vs. fitted values for all foundation models in one plot.

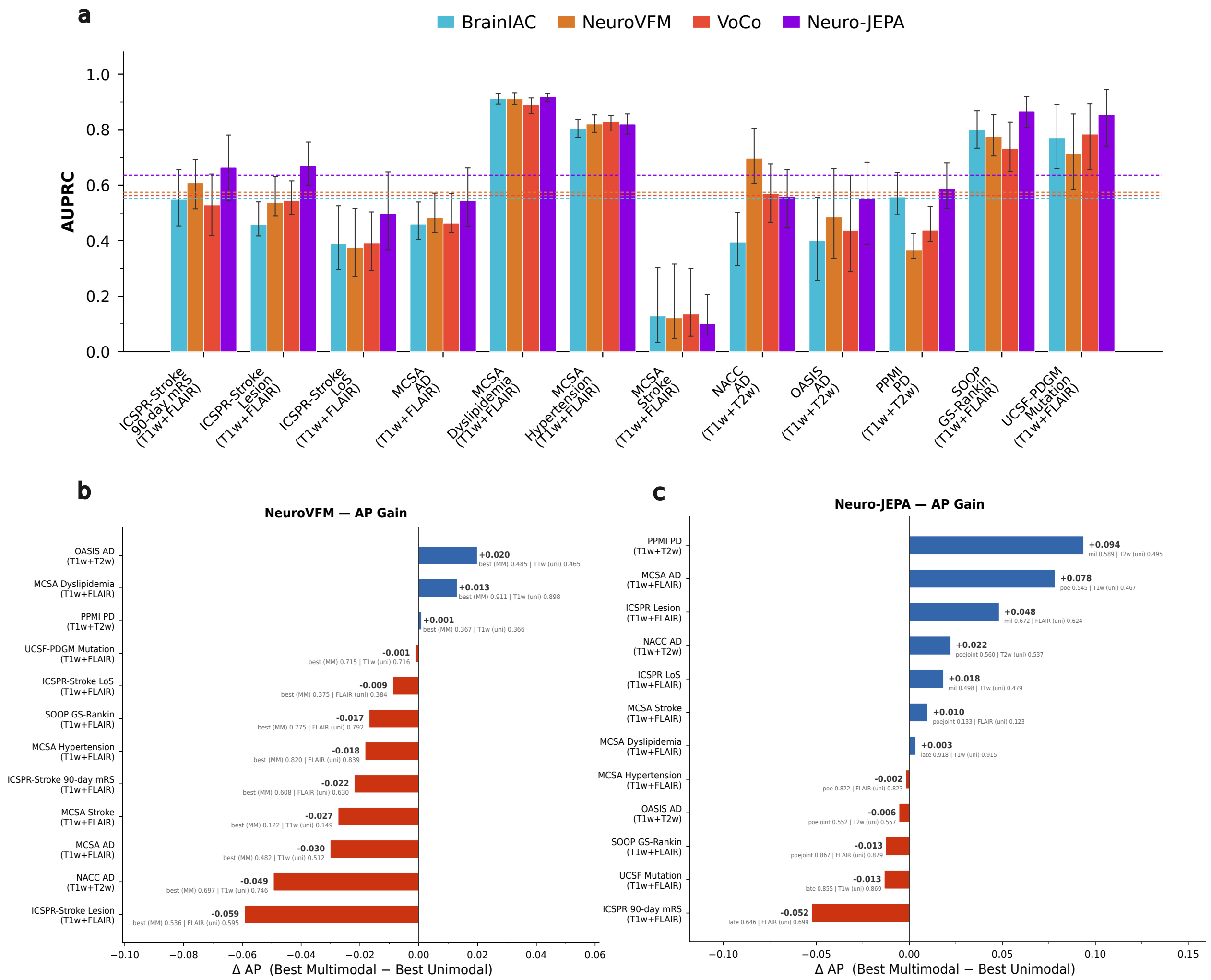

**Extended Data Fig. 5. Multimodal Performance and Gain Over Unimodal on AUPRC.** We report AUPRC on multimodal performance when two different modalities are combined and multimodal performance gain over unimodal defined as the difference between best multimodal combination and best unimodal performance. **a,** Multimodal performance on AUPRC across selected tasks on public datasets for all four compared foundation models. The result is reported by best performance multimodal fusion method among five different methods. Dotted horizontal line present average performance across tasks, where the result shows our model outperforms other foundation models with a large margin. **b,c,** Multimodal gain on AUPRC for best previous foundation model (NeuroVFM) and ours. The result shows that our model present better performance gain under multimodal fusion over previous model.

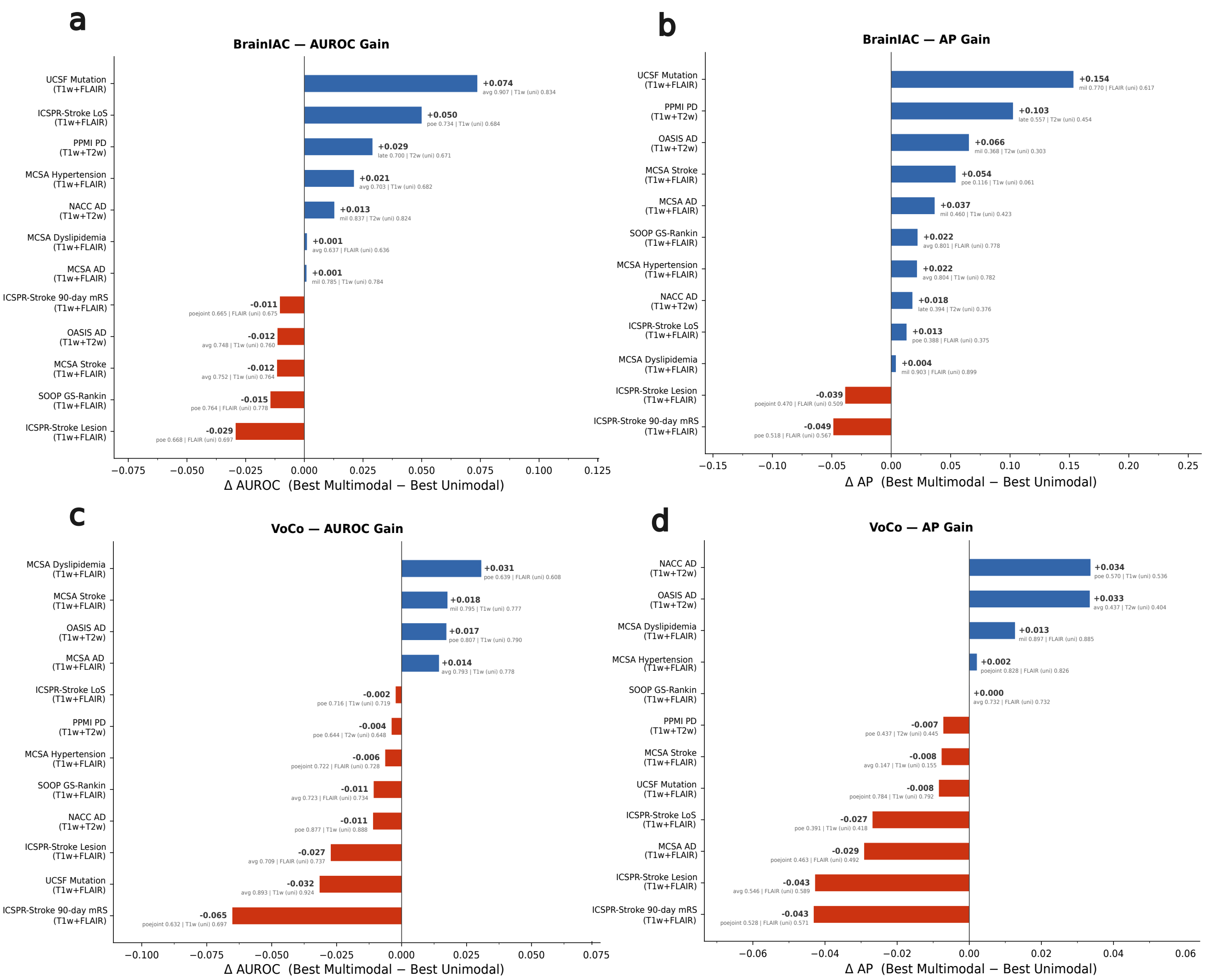

**Extended Data Fig. 6. Mutlimodal Gain Over Unimodal for BrainIAC and VoCo.** The difference between best multimodal fusion vs. best unimodal performance on AUROC and AUPRC. **a,b,** AUROC and AUPRC multimodal performance gain on the difference for BrainIAC. **c,d,** AUROC and AUPRC multimodal performance gain on the difference for VoCo.

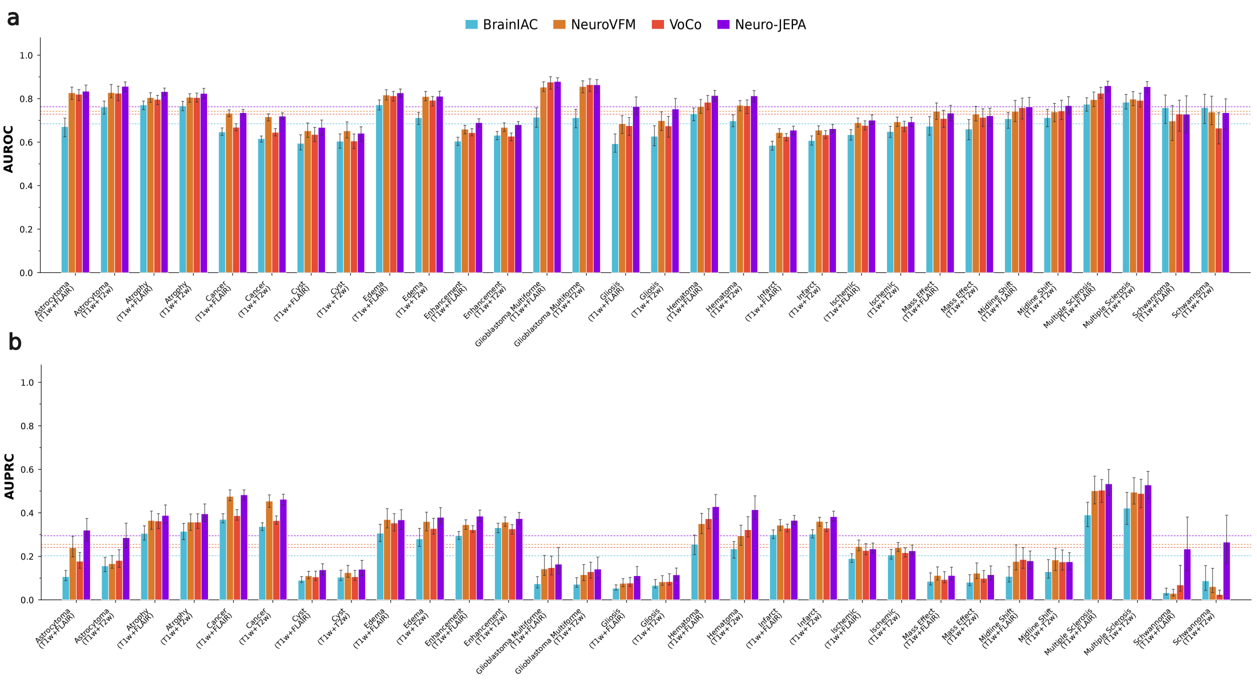

**Extended Data Fig. 7. Multimodal Performance on BIND-MGH.** We report AUROC and AUPRC for multimodal performance on all models when two different modalities are combined. The result is reported by best performance multimodal fusion method among five different methods. Dotted horizontal line present average performance across tasks, where the result shows our model outperforms other foundation models with a large margin. **a,** AUROC for different tasks across evaluated models **b,** AUPRC for different tasks across evaluated models.

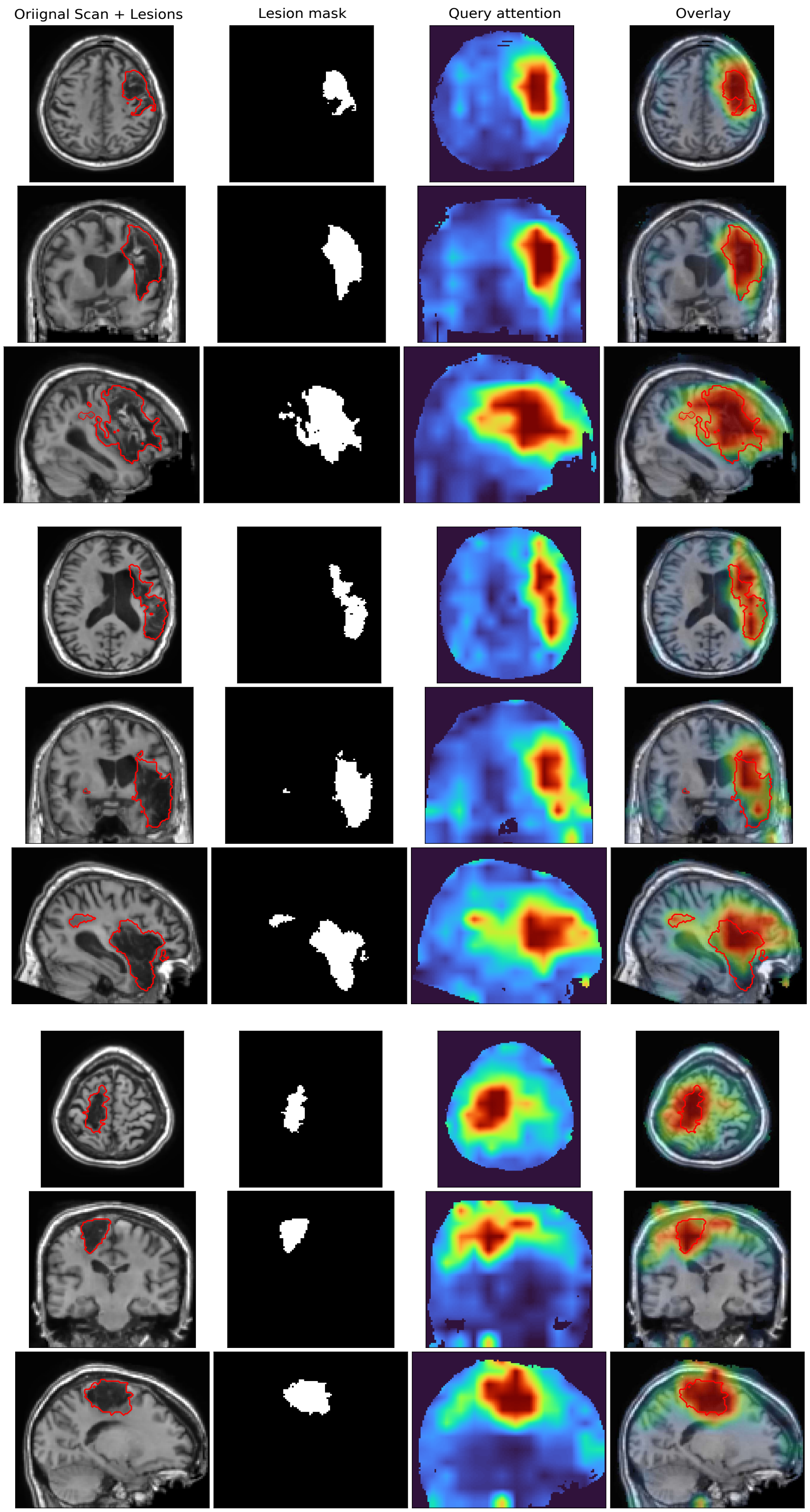

**Extended Data Fig. 8.** **Attention Visualization - Stroke Lesions.**

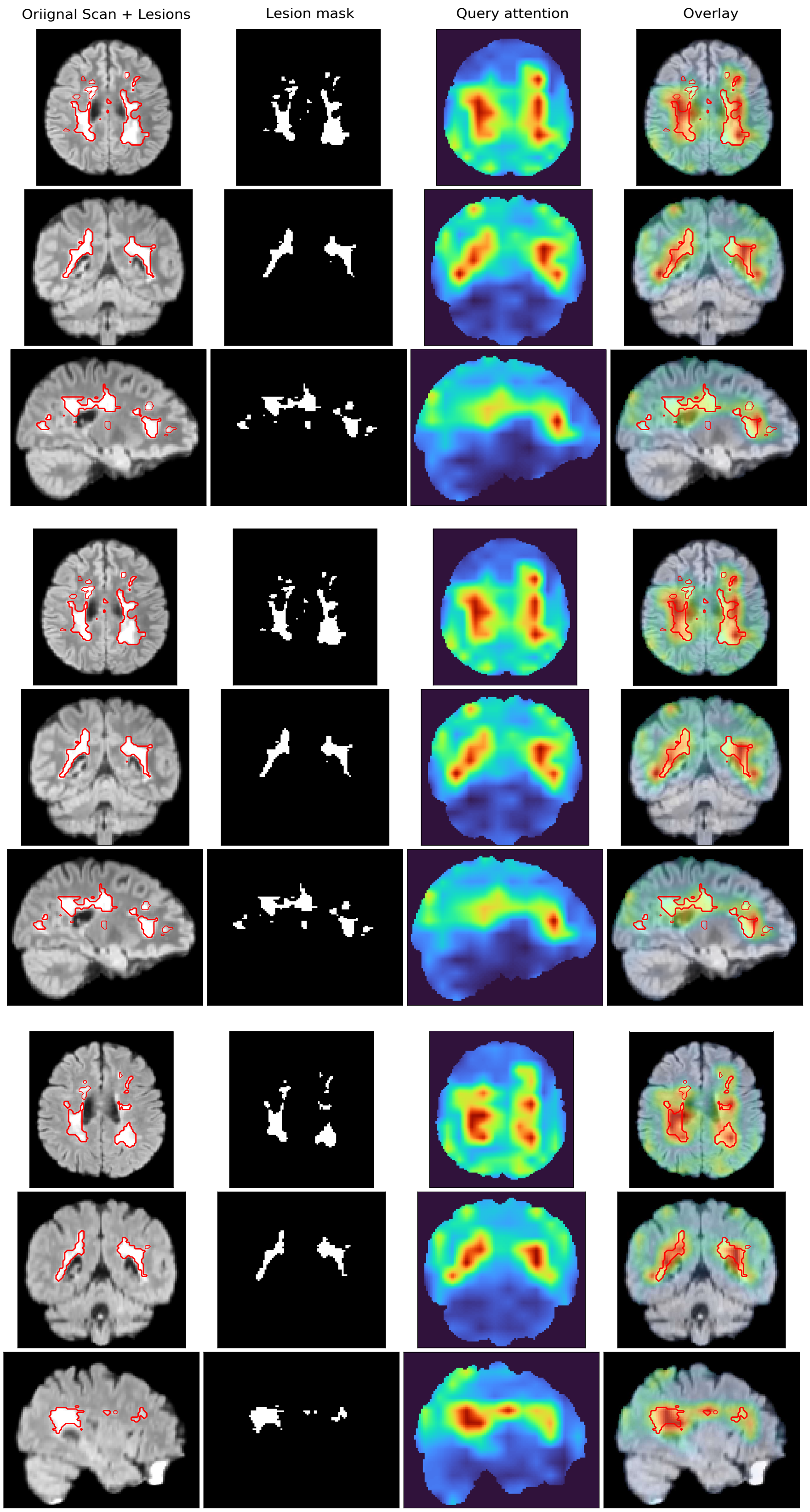

**Extended Data Fig. 9.** **Attention Visualization - Multiple Sclerosis Lesions.**

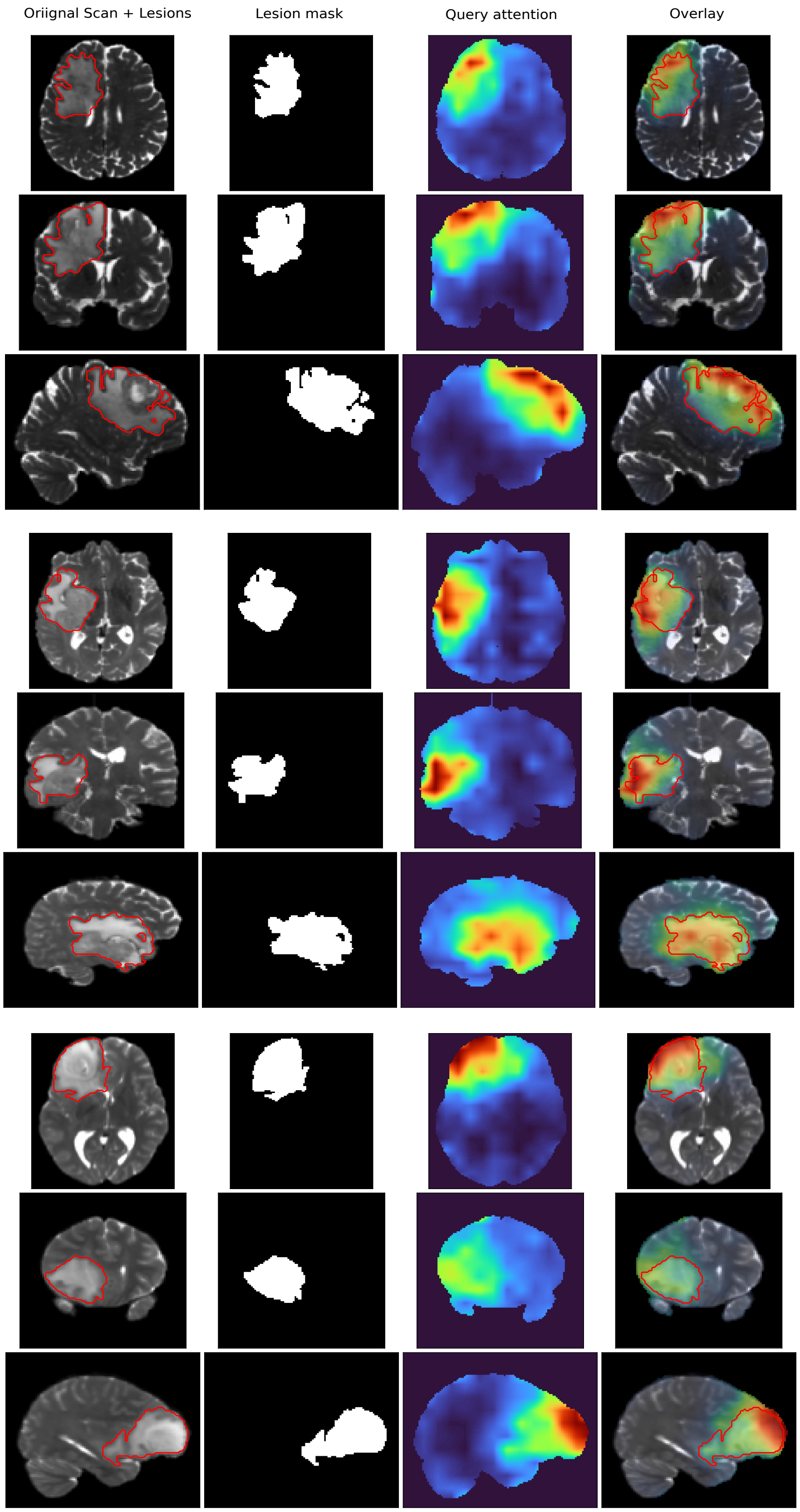

**Extended Data Fig. 10.** **Attention Visualization - Gliobastoma Lesions.**

## Supplementary Files

This is a list of supplementary files associated with this preprint. Click to download.

- Supplementary.pdf
